# Phosphorylation independent eIF4E translational reprogramming of selective mRNAs determines tamoxifen resistance in breast cancer

**DOI:** 10.1038/s41388-020-1210-y

**Published:** 2020-02-17

**Authors:** Chun Gong, Ho Tsoi, Ka Chun Mok, Jenny Cheung, Ellen P. S. Man, Kazunari Fujino, Ashely Wong, Eric W. F. Lam, Ui-Soon Khoo

**Affiliations:** 1 Department of Pathology, Li Ka Shing Faculty of Medicine, The University of Hong Kong, Hong Kong SAR, Hong Kong; 2 Department of Surgery and Cancer, Imperial College London, Hammersmith Hospital Campus, London, UK

**Keywords:** Tumour biomarkers, Breast cancer, Cell signalling

## Abstract

Eukaryotic translation initiation factor 4E (eIF4E) selectively promotes translation of mRNAs with atypically long and structured 5′-UTRs and has been implicated in drug resistance. Through genome-wide transcriptome and translatome analysis we revealed eIF4E overexpression could promote cellular activities mediated by ERα and FOXM1 signalling pathways. Whilst eIF4E overexpression could enhance the translation of both ERα and FOXM1, it also led to enhanced transcription of FOXM1. Polysome fractionation experiments confirmed eIF4E could modulate the translation of ERα and FOXM1 mRNA. The enhancement of FOXM1 transcription was contingent upon the presence of ERα, and it was the high levels of FOXM1 that conferred Tamoxifen resistance. Furthermore, tamoxifen resistance was conferred by phosphorylation independent eIF4E overexpression. Immunohistochemistry on 134 estrogen receptor (ER^+^) primary breast cancer samples confirmed that high eIF4E expression was significantly associated with increased ERα and FOXM1, and significantly associated with tamoxifen resistance. Our study uncovers a novel mechanism whereby phosphorylation independent eIF4E translational reprogramming in governing the protein synthesis of ERα and FOXM1 contributes to anti-estrogen insensitivity in ER^+^ breast cancer. In eIF4E overexpressing breast cancer, the increased ERα protein expression in turn enhances FOXM1 transcription, which together with its increased translation regulated by eIF4E, contributes to tamoxifen resistance. Coupled with eIF4E translational regulation, our study highlights an important mechanism conferring tamoxifen resistance via both ERα dependent and independent pathways.

## Introduction

Breast cancer is the most common female cancer worldwide. The estrogen receptor (ER) signalling pathway is the fundamental proliferative pathway in breast cancer, which upon its activation by binding with estrogen, activates target gene transcription and cell growth. Up to 70% of patients with breast cancer are estrogen receptor positive (ER^+^) and can benefit from anti-estrogen therapy, Tamoxifen being most commonly prescribed for ER^+^ premenopausal breast cancer in order to prevent cancer relapse or metastases [1]. However, resistance remains an outstanding problem with resistance occurring in up to 30% of patients [2] who often progress to metastases and death. Although tremendous efforts have been made to understand the mechanism underlying tamoxifen resistance, there remain areas not fully understood.

Translation is the last process in the flow of genetic information, and its precise control provides an immediate and rapid response to cope with physiological changes. In eukaryotes, translation initiation is the most regulated step. Aberrant function of components of the translation machinery underlies a variety of human diseases including breast cancer [3]. Precise regulation of protein translation is important for the activity of oncogenes and tumour suppressor genes which contribute to oncogenesis [4, 5] as well as the development of drug resistance, such as to trastuzumab [6], and to a variety of PI3K-AKT-mTOR inhibitors [7–9].

Eukaryotic translation initiation factor 4E (eIF4E) is the least abundant initiation factor and is rate limiting for translation initiation [10, 11]. Early studies showed that overexpression of eIF4E facilitates the translation of mRNAs containing structural repeats at the 5′ untranslated region (5′UTR) in vivo [12]. The average well-translated mRNA has a 5′UTR of 20–50 nucleotides. Around 10% of cellular mRNAs contain atypically long 5′UTR and many of these encode proto-oncogenes, anti-apoptotic proteins and growth factors. A long 5′UTR and GC rich sequence tends to form a stable secondary structure. It has been shown that translation of mRNA with long 5′UTR is often sensitive to expression level of eIF4E [13]. Indeed, overexpression of eIF4E in cells can promote translation of certain mRNAs involved in cell proliferation, apoptosis and tumour progression, such as cyclin D1, ornithine decarboxylase, c-myc, vascular endothelial growth factor (VEGF) and FGF-2. Conversely, knockdown of eIF4E can suppress the translation of VEGF, cyclin D1, c-myc, Bcl-2 and Bcl-xL [14–17].

We hypothesized that eIF4E may be an important factor in determining tamoxifen resistance. Using an eIF4E overexpressing model system on tamoxifen sensitive cell lines we investigated whether genome-wide transcriptome and translatome analysis could identify specific translational reprogramming pathways that targets anti-estrogen and ER activities. The estrogen pathway and FOXM1 signalling pathways were consistently identified as amongst the top ten altered pathways in eIF4E overexpressing cells. Interestingly, whilst eIF4E overexpression enhanced the translation of both ERα and FOXM1, it also resulted in enhanced transcription of FOXM1. Polysome fractionation experiments confirmed that eIF4E overexpression could significantly increase protein expression of ERα and FOXM1. The enhancement of FOXM1 mRNA expression in eIF4E overexpressed cells on the other hand, was contingent upon the presence of ERα, and it was this enhanced FOXM1 that conferred Tamoxifen resistance. Consistent with this hypothesis, using Tissue Microarray sections of breast cancer samples, we confirmed that elevated eIF4E expression was significantly associated with ERα and FOXM1 protein expression as well as with tamoxifen resistance. These findings provide better insight into how eIF4E expression contributes towards anti-estrogen insensitivity in ER^+^ breast cancer.

## Results

### Overexpression of eIF4E modulated the expressions of ERα and FOXM1 in breast cancer

**Fig. 1:**
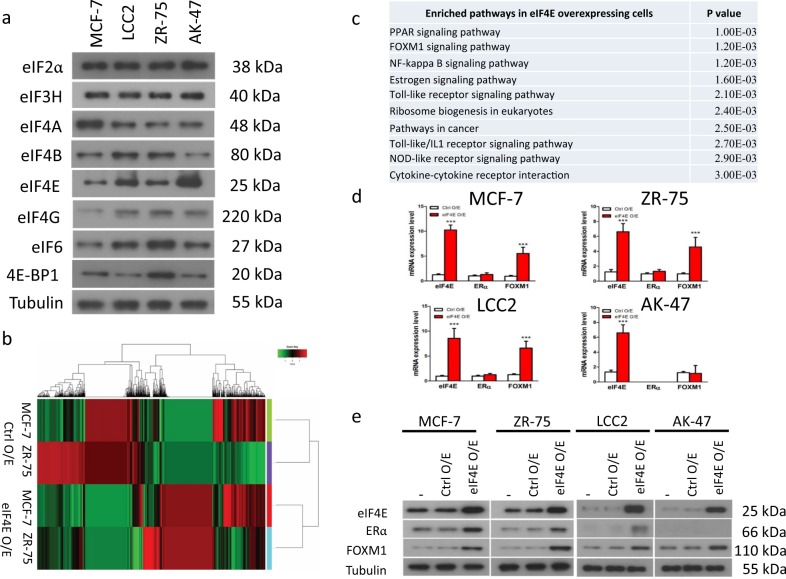
Overexpression of *eIF4E* could modulate the molecular pathways mediated by ERα and FOXM1. **a** Comparing the protein expression of a panel of translation regulators between tamoxifen sensitive (MCF-7 and ZR-75) and resistant cells (LCC2 and AK-47). Western blotting was used to determine the expression of the indicated protein candidates. Tubulin was used as the loading control. **b** Overexpression of *eIF4E* in MCF-7 and ZR-75 breast cancer cells could induce distinctive molecular alterations in polysomes. The cells were transfected with *pCMV6_eIF4E* for 72 h. RNA sequencing was performed to determine the effect of eIF4E overexpression on mRNA profile in the polysome. Heatmap was used to show the profiles of the molecular features. **c** Top 10 molecular pathways being enriched by overexpression of eIF4E. KEGG pathway analysis was performed to identify pathways which were potentially enriched in eIF4E overexpressing breast cancer cells. **d** The effect of eIF4E overexpression on the mRNA levels of eIF4E, ERα and FOXM1 in MCF-7, ZR-75, LCC2 and AK-47 cells. qPCR was performed to determine the levels of the corresponding mRNAs. Actin was used as the internal control. Results were expressed as mean ± s.d. from three independent experiments. Student *t-*test was employed. *** represents *P* < 0.001. **e** The effect of eIF4E overexpression on the protein levels of eIF4E, ERα and FOXM1 in MCF-7, ZR-75, LCC2 and AK-47 cells. The cells were transfected with 2 µg of *pCMV6_eIF4E* (eIF4E O/E) or *pCMV6* (Ctrl O/E). Whole cell lysates were harvested after 72 h posttransfection. Western blot was performed. Tubulin was used as the loading control.

Eukaryotic translation initiation factors have been shown to be associated with poor outcome in different cancers and with drug resistance [18, 19]. To confirm that eIF4E is the translation initiation factor associated with tamoxifen resistance, we compared the protein expression of the various translational factors in tamoxifen sensitive (MCF-7 and ZR-75) versus tamoxifen-resistant (LCC2 and AK-47) cell lines. Western blot confirmed high eIF4E expression was consistently found in resistant cell lines (Fig. 1a), suggesting that overexpression of eIF4E might contribute to tamoxifen resistance in breast cancer.

To investigate the underlying mechanisms through which eIF4E may modulate tamoxifen response, genome-wide transcription and translation mRNA profiling was performed to determine the effect of eIF4E overexpression on cellular activities in ER^+^ breast cancer, comparing expression patterns of MCF7 and ZR-75, with either *eIF4E* overexpression or knockdown. Overexpression of *eIF4E* yielded a distinctive gene expression profile, for both total mRNA profiling (Fig. S1a; Supplementary Table 1) and polysomal mRNA profiling (Fig. 1b; Supplementary Table 2). The various altered cellular pathways enriched by eIF4E overexpression, as identified by KEGG pathway analysis, are as listed for transcriptome profile (Supplementary Table 3) and for translatome profile (Supplementary Table 4). Interestingly, the estrogen signalling pathway and the FOXM1 transcription pathway, previously reported to be implicated in tamoxifen resistance [20, 21], were consistently identified as amongst the top ten pathways for both total mRNA and polysomal mRNA profiles (Figs. 1c and S1b), the findings at translatomic level being reflected at transcriptomic level. These results suggest that eIF4E overexpression modulate estrogen and FOXM1 signalling pathways in breast cancer, which were thus chosen for further study.

Although estrogen and FOXM1 signalling pathways were enriched, considering that eIF4E modulates translation rather than transcription, increased ERα and FOXM1 mRNA expression was not expected. Validation by qPCR however showed that eIF4E overexpression could enhance mRNA expression of FOXM1 but not of ERα in MCF-7, ZR-75 and LCC2 cells. Neither did it affect FOXM1 mRNA expression in AK-47, an ERα negative cell line (Fig. 1d), suggesting that FOXM1 mRNA modulation by eIF4E may be ERα dependent. Western blot on the other hand, showed that eIF4E overexpression enhanced protein expression of FOXM1 in all cell lines and ERα in all ERα positive cell lines (Fig. 1e), suggesting that overexpression of eIF4E can modulate the translation of both ERα and FOXM1.

### Overexpression of eIF4E favoured the translation of ERα and FOXM1

Translation initiated by the cap-dependent pathway can be affected by structures at the 5′UTR [22]. The translation of mRNAs which contain atypically long and structured 5′-UTRs (several hundred to a thousand nucleotides long) are often sensitive to expression levels of eIF4E via the cap-dependent pathway [22], while the translation of mRNAs which have short and unstructured 5′-UTRs (usually 20 to 50 nucleotides) are usually not eIF4E-sensitive and often encode for housekeeping genes such as *GAPDH* (Fig. S2a) and β-*actin* (Fig. S2b) [23].

To evaluate the possibility of *ERα* and *FOXM1* being 4E-sensitive, their 5′-UTR length and structure were analysed and predicted in silico. Comparison was made with eIF4E-sensitive genes *MYC* (Fig. S2c) and *cyclin D1* (Fig. S2d) and non-eIF4E-sensitive genes *GAPDH* and *β-actin*. The complexity of the secondary structure of the 5′-UTR was determined by the length and percentage of GC content. According to the NCBI database, *ERα* and *FOXM1* have a 5′-UTR of 243 and 284 base pairs (bp) respectively, which are much longer when compared with the 5′-UTR of *GAPDH* (102 bp) and *β*-*actin* (84 bp). By software “RNAfold”, the 5′-UTR of *ERα* (Fig. S2e) and *FOXM1* (Fig. S2f) were folded into a more complex and stable secondary structure characterized by multiple hydrogen bonding between paired bases, when compared with that of *GAPDH* (Fig. S2a) and *β*-*actin* (Fig. S2b); furthermore, the minimum free energy levels of the 5′-UTR of *ERα* and *FOXM1* were higher (−111.98 and −128.78 kcal/mol respectively), compared with that of *GAPDH* and *β*-*actin* (−42.32 and −15.92 kcal/mol respectively) and comparable to that reported for other 4E-sensitive mRNAs such as *MYC* and *cyclin D1* (Fig. S2c, d). Therefore, it is likely the translation of ERα and FOXM1 mRNAs is sensitive to eIF4E expression levels in the cell.

**Fig. 2:**
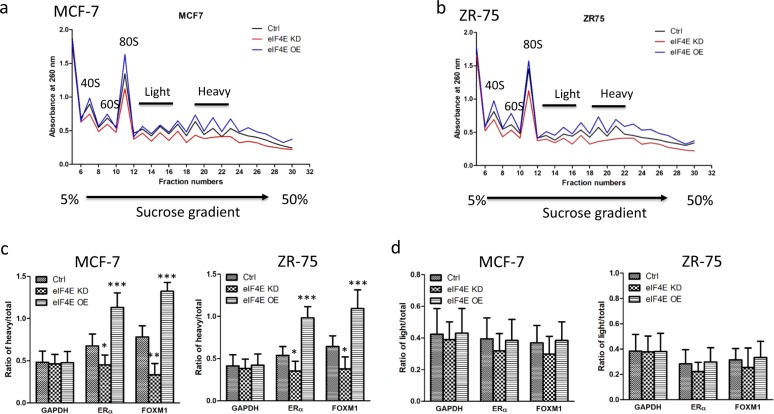
Alteration of eIF4E expression could modulate the translational behaviour of ERα and FOXM1. Polysome fractionation was performed on (**a**) MCF-7 and (**b**) ZR-75 cells. Overexpression of *eIF4E* was mediated by transfection of 4 μg of *pCMV6_eIF4E* while knockdown of *eIF4E* was mediated by transfection of 120 pmol of eIF4E siRNA#1. Cells were harvested after 72 h posttransfection. The absorbance at 260 nm in each of the fractions was monitored. Fractions 13–17 were regarded as the light fractions while fractions 19–23 were regarded as heavy fractions (polysome). Ctrl represents untreated sample, eiF4E KD represents *eIF4E* knockdown, eIF4E OE represents *eIF4E* overexpression. **c** Alteration of *eIF4E* expression could alter the abundance of ERα and FOXM1 mRNAs in the heavy fractions in MCF-7 and ZR-75. **d** Alteration of *eIF4E* expression did not affect the abundance of ERα and FOXM1 mRNAs in the light fractions in MCF-7 and ZR-75. qPCR was performed to detect the presence of the mRNAs in the heavy and light fractions. Expression level of the mRNAs in the heavy fraction and the light fraction was compared relative to the mRNAs’ expression level in unfractionated sample. Results were expressed as mean ± s.d. from three independent experiments. Student *t-*test was employed to determine if there was statistical significant between Ctrl O/E and eIF4E O/E groups. “*”, “**” and “***” indicate a statistical significance with *P* < 0.05 and *P* < 0.001.

To confirm our translatomic findings that *ERα* and *FOXM1* mRNAs may be eIF4E-dependent, polysome fractionation experiments were carried out to separate the actively translating mRNAs that are associated with multiple ribosomes (polysomal, heavy fraction) from the inactively translating mRNAs that are bounded with no or few ribosomes (monosomal, light fraction) in the *eIF4E* overexpression and *eIF4E* knockdown cells. The results showed that modulation of *eIF4E* expression could alter the translation profile; overexpression of *eIF4E* heightened the peak signals detected in the heavy fraction while knockdown of *eIF4E* appeared to disrupt the polysomes in the heavy fraction, as indicated by the flattening of peaks (Fig. 2a, b). qPCR was used to detect the presence of *ERα*, *FOXM1* and *GAPDH* mRNAs in the heavy and light fractions, which were normalized against total amount (unfractionated) of the corresponding genes. Comparing the level of *ERα* and *FOXM1* mRNAs in the heavy fraction, we found that knockdown of *eIF4E* reduced the levels of both *ERα* and *FOXM1* while overexpression of *eIF4E* increased the levels of both *ERα* and *FOXM1* in the heavy fraction. The levels of *GAPDH* however remained unaffected (Fig. 2c). In the light fraction however, both knockdown and overexpression of *eIF4E* had minimal impact on *ERα* and *FOXM1* (Fig. 2d). The efficiency of protein translation in the polysomes (heavy fraction) is higher than that in the pre-polysomes (light fraction). Thus, the increased *ERα* and *FOXM1* transcript translation efficiency observed with *eIF4E* overexpression can account for the increased ERα and FOXM1 proteins being translated.

### eIF4E modulated protein expression of ERα and FOXM1, and altered FOXM1 mRNA expression

**Fig. 3:**
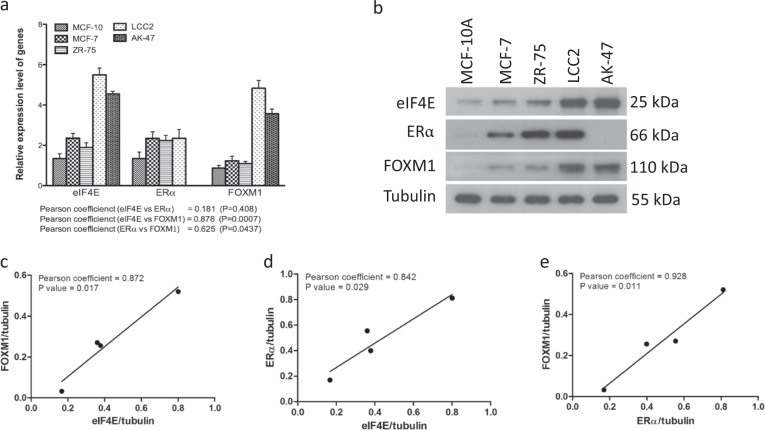
The correlation between eIF4E, ERα and FOXM1 on mRNA and protein levels in vitro. **a** The mRNA expressions of eIF4E, ERα and FOXM1 in MCF-10A, MCF-7, ZR-75, LCC2 and AK-47 cell lines were determined by qPCR. Actin was used as the internal control. Results were expressed as mean ± s.d. from three independent experiments. Linear regression model was employed to determine the correlation. **b** Protein expressions of eIF4E, ERα and FOXM1 in the indicated cell lines were determined by western blot. Tubulin was used as the loading control. Correlation between the expression levels of (**c**) eIF4E and FOXM1, (**d**) eIF4E and ERα and (**e**) ERα and FOXM in MCF-10A, MCF-7, ZR-75 and LCC2 was determined. AK-47 is an *ERα* negative cell line, hence correlation were only made from 4 cell lines. Band intensity was measured by ImageJ from three independent experiments. The expression of protein candidates was relative to tubulin. Each of the dots represented one cell line. Linear regression model was employed to determine the correlation. *P* < 0.05 was regarded as statistical significance.

Estrogen receptor-alpha (ERα) has been reported to be directly involved in transcriptional regulation of FOXM1 [20, 21], which may contribute towards tamoxifen resistance [20]. To determine whether eIF4E might play a role in mediating tamoxifen resistance via the estrogen-dependent pathway, constitutive expression of eIF4E, ERα and FOXM1 were correlated in a panel of breast cancer cell lines; tamoxifen sensitive MCF-7 and ZR-75, and tamoxifen-resistant LCC2 (ER^+^) and AK-47 (ER^−^) cells, together with non-neoplastic breast epithelial cell MCF-10A which expresses low levels of all three genes. The results from qPCR demonstrated that at mRNA level, *eIF4E* was significantly correlated with *FOXM1* (*P* = 0.0007; Fig. 3a) but not with *ERα* (*P* = 0.408; Fig. 3a). Correlation between *ERα* and *FOXM1* was observed (*P* = 0.0437; Fig. 3a). Their expression at protein level was examined by western blot (Fig. 3b), with band intensities quantified using ImageJ. The results showed that eIF4E was significantly correlated with FOXM1 (*P* = 0.017; Fig. 3c) and with ERα (*P* = 0.029; Fig. 3d). Correlation between ERα and FOXM1 was also observed (*P* = 0.011; Fig. 3e). These results support our hypothesis that ERα and FOXM1 translation are sensitive to upregulated eIF4E expression and are also in keeping with our observation that eIF4E overexpression resulted in enhanced mRNA expression of FOXM1 but not ERα. Moreover, both eIF4E and FOXM1 were expressed at higher levels in tamoxifen-resistant cells LCC2 and AK-47 compared with their parental tamoxifen sensitive cells MCF-7 and ZR-75, at both mRNA and protein levels (Fig. 3a, b). Interestingly, whilst correlation between eIF4E and FOXM1 was observed at both transcription and translation levels, correlation between eIF4E and ERα was only at translation level.

**Fig. 4:**
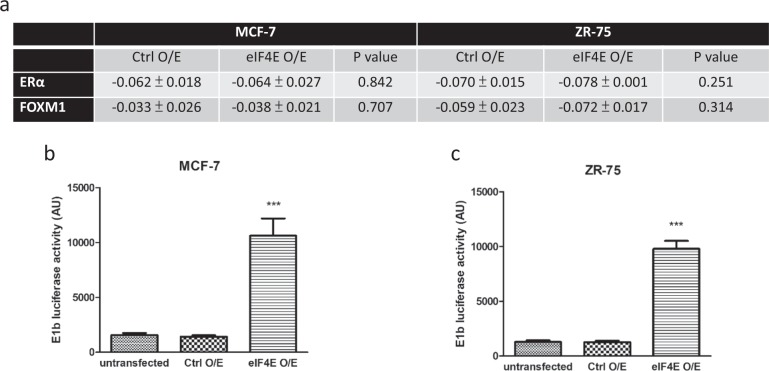
Overexpression of eIF4E did not alter the mRNA stability of ERα and FOXM1 but enhanced ERα transcriptional activity. **a** Overexpression of eIF4E did not alter the mRNA stability of ERα and FOXM1. The cells were treated with 5 µg/mL of ActD for 0 hour and 24 h. qPCR was employed to determine the expression of the candidate mRNA. The formula C_24_ = C_0_ e^−kt^ was employed to determine the decay constant (**k**) of each of the candidate mRNA. C_24_ and C_0_ were the relative expression of the mRNA at 24-hour and 0-hour. The length of the experiment was t. Overexpression of eIF4E could enhance the transcriptional activity of ERα in (**b**) MCF-7 and (**c**) ZR-75 cells. Luciferase reporter assay was employed. The promoter region contained estrogen receptor response element. The luciferase activity positively correlated with the transcription activity of ERα. Results were expressed as mean ± s.d. from three independent experiments. Student *t-*test was used to determine statistical significance.

Given this unexpected increased transcription of FOXM1 in eIF4E overexpressing cells, it was necessary to exclude the possibility eIF4E overexpression might stabilize transcripts, thus preventing their degradation. RNA stability assay was performed to determine whether overexpression of eIF4E would affect *ERα* and *FOXM1* mRNA. From the our results, the k value which represents decay constant, was similar for both of mRNA in control and eIF4E overexpressing cells (Fig. 4a), indicating that overexpression of *eIF4E* should not affect the stability of *ERα* and *FOXM1* mRNA.

### eIF4E modulated the transcription of FOXM1 in ER^+^ cells

Since our in vitro expression studies found eIF4E overexpression altered the expression of FOXM1 at both transcription and translation levels, but that of ERα only at translation level, we next examined the effect of gene knockdown on the expression of the other two genes at both mRNA and protein levels. Knockdown of *eIF4E* by two independent siRNAs in MCF-7 and ZR-75, could reduce the expression of ERα at protein level and expression of FOXM1 at both mRNA and protein levels (Fig. S3). Knockdown of *ERα* could reduce the expression of FOXM1 at both mRNA and protein levels but did not alter the expression of eIF4E (Fig. S4). Knockdown of *FOXM1* neither altered *eIF4E* nor *ERα* at mRNA or protein levels (Fig. S5). Results were similar for ER^+^ tamoxifen-resistant cell LCC2 (Fig. S6), and for ER^−^ tamoxifen-resistant cell AK-47 (Fig. S7). Taken together with the eIF4E overexpression data, these findings suggest that whilst eIF4E modulation can alter both ERα and FOXM1 protein expression, it is the changes in ERα protein expression that account for modulation in FOXM1 transcription. Since it has been shown that ERα can regulate FOMX1 transcription [20, 21], our findings suggest that whilst eIF4E overexpression upregulates both ERα and FOXM1 protein expression, it is the increased ERα protein expression which in turn enhances FOXM1 transcription. To confirm this, E1b luciferase activity assay was employed, which indeed confirmed that overexpression of eIF4E could enhance the transcription activity of ERE (Fig. 4b, c).

### eIF4E overexpression altered tamoxifen response in breast cancer and modulated this response via FOXM1

**Fig. 5:**
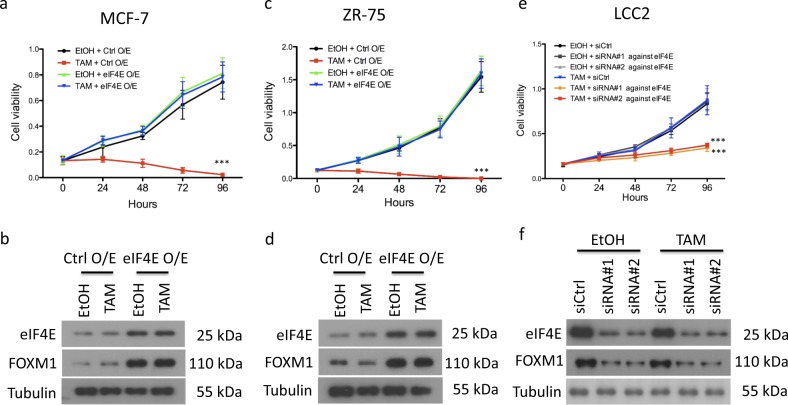
Alteration of eIF4E expression could modulate the response to tamoxifen in ER^**+**^ breast cancer cells. Overexpression of *eIF4E* could induce tamoxifen resistance in (**a**) MCF-7 and (**c**) ZR-75 cells. 0.5 μg of *pCMV6* was used as control while overexpression of *eIF4E* was mediated by transfection of 0.5 μg of *pCMV6_eIF4E*. The effect on eIF4E and FOXM1 expression in (**b**) MCF-7 and (**d**) ZR-75 was confirmed by western blot. Tubulin was used as the loading control. The cells were treated with either ethanol (EtOH) or 4 µM of tamoxifen (TAM). **e** Knockdown of *eIF4E* could reverse tamoxifen resistance in LCC2 cells. Knockdown of *eIF4E* was mediated by transfection of 20 pmol of siRNA#1 or siRNA#2. 20 pmol of non-targeting siRNA (siCtrl) was used. **f** The knockdown efficiency of the siRNAs on eIF4E and FOXM1 expression in LCC2 was determined by western blot. Tubulin was used as the loading control. The cells were treated with either EtOH or 4 µM of tamoxifen. MTT assay was employed to determine the cell viability. Results were expressed as mean ± s.d. from three independent experiments. “***” indicates both time and the treatment significantly affect the cell viability with a statistical significance with *P* < 0.001 by two way ANOVA.

MCF-7 and ZR-75 are ERα^+^ tamoxifen sensitive cell lines [24, 25], exhibiting reduced cell viability on tamoxifen treatment. However, once *eIF4E* was overexpressed in these cell lines, the effect of tamoxifen on cell viability was compromised (Fig. 5a–d). Conversely, knockdown of *eIF4E* rendered LCC2, become more sensitive to tamoxifen (Fig. 5e, f). These results are supportive of our hypothesis that modulation of eIF4E expression can alter tamoxifen response.

**Fig. 6:**
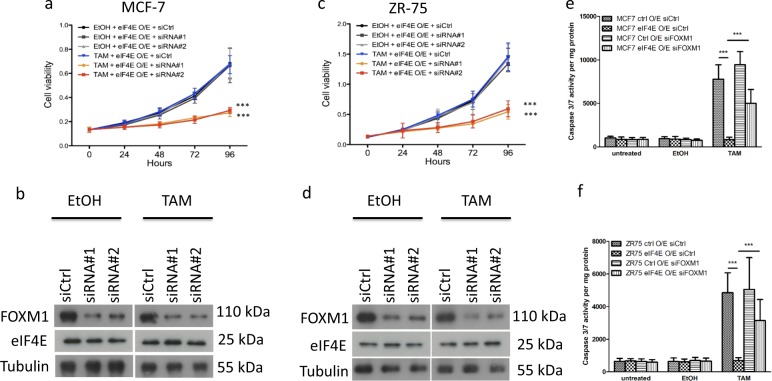
Knockdown of *FOXM1* could compromise the effect of eIF4E overexpression on tamoxifen resistance. Stable eIF4E overexpressing (**a**) MCF-7 and (**c**) ZR-75 cell lines were used. Knockdown of *FOXM1* was mediated by transfection of 50 pmol of the corresponding siRNA#1 or siRNA#2 in the stable eIF4E overexpressing cells. 50 pmol of non-targeting siRNA (siCtrl) was used as control. The cells were treated with either EtOH or 4 µM of tamoxifen. MTT assay was employed to determine the cell viability. Results were expressed as mean ± s.d. from three independent experiments. “***” indicates both time and siRNA treatments significantly affect the cell viability with a statistical significance with *P* < 0.001 by two way ANOVA. The knockdown effect of *FOXM1* by the siRNAs and the expression of eIF4E in (**b**) MCF-7 and (d) ZR-75 was confirmed by western blot. Tubulin was used as the loading control. Knockdown of *FOXM1* could resume the effect of tamoxifen on caspase activation in eIF4E overexpressing cells. Knockdown of *FOXM1* was mediated by transfection of 50 pmol of the corresponding siRNA#1. Caspase 3/7 activity assay was performed on (**e**) MCF-7 eIF4E O/E and (**f**) ZR-75 eIF4E O/E cells. “***” represents *P* < 0.001. Student *t-*test was employed to determine the statistical difference between two groups.

We have shown that upregulation of eIF4E enhances expression of both ERα and FOXM1 protein. Enhanced ERα expression would be expected up to a certain extent, to render cells more sensitive to tamoxifen. Hence it would be the upregulated FOXM1 expression that more likely contributes to tamoxifen resistance. Indeed it has been reported that FOXM1 is a key mediator of mitogenic functions of ERα and oestrogen in breast cancer cells, contributing towards tamoxifen resistance [20]. To determine whether FOXM1 would be required to modulate tamoxifen resistance in eIF4E overexpressed cells, we knocked down *FOXM1* in stably *eIF4E* overexpressing MCF-7 and ZR-75 cells. The results showed that knockdown of *FOXM1* could compromise the tamoxifen resistance conferred by overexpression of *eIF4E* in MCF-7 (Fig. 6a, b) and ZR-75 cells (Fig. 6c, d), by recovering caspase activation mediated by tamoxifen in eIF4E overexpressing cells (Fig. 6e, f). On the other hand, knockdown of either *MYC* or *cyclin D1* failed to resume tamoxifen response in eIF4E overexpressing cells but reduced the degree of cell proliferation (Fig. S8 and Fig. S9). These findings confirm the important role of FOXM1 on conferring tamoxifen resistance in eIF4E overexpressing cells.

### Expression of eIF4E correlated with FOXM1 expression and tamoxifen resistance in vivo

**Fig. 7:**
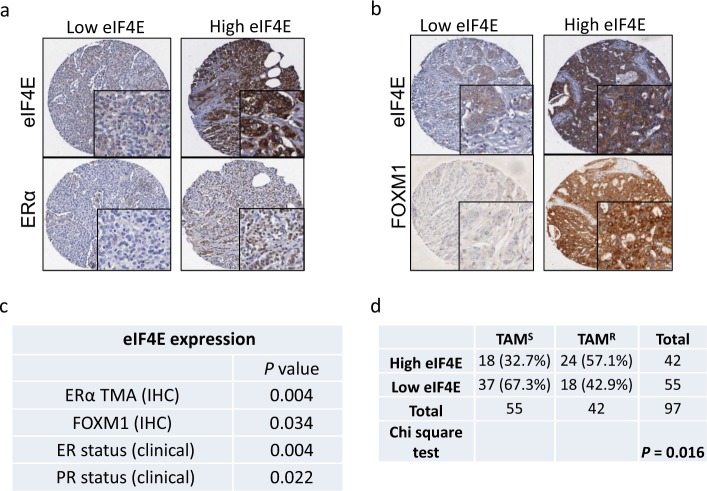
Correlation eIF4E with ERα and FOXM1 protein expression levels in vivo. **a** Representative photomicrographs of high and low expression of eIF4E and ERα in breast tumours in TMA achieve. **b** Representative photomicrographs of high and low expression of eIF4E and FOXM1 in breast tumours in TMA achieve. IHC was employed to stain the target protein in the 134 cases of ER+ve tumour tissues. **c** eIF4E scores were correlated with clinically reported ER and PR status obtained from pathology reports. eIF4E was positively correlated with ERα and FOXM1 was observed. Chi-square test was performed to determine the significance. **d** High expression of eIF4E was associated with tamoxifen resistance. There were 97 breast cancer cases, among them 55 being tamoxifen sensitive and 42 being tamoxifen-resistant. Chi-square test was performed to determine the significance.

Reports have shown that eIF4E is overexpressed in different types of cancers [26], including breast cancer. To confirm our in vitro findings, immunohistochemistry (IHC) for eIF4E, ERα and FOXM1 was performed on 134 primary breast cancer samples in Tissue MicroArray (TMA) (Fig. 7a, b). Indeed, we observed significant direct correlation between eIF4E and ERα, as well as between eIF4E and FOXM1 (*P* < 0.05; Fig. 7c). The expression level of eIF4E was also significantly associated with the clinically reported ER and PR (progesterone receptor) status obtained from pathology reports of the cases (Fig. 7c), PR being estrogen-dependent and its status indicating the intact estrogen-response pathway [27].

Examining the relationship of eIF4E protein expression with tamoxifen response in our TMA samples of 97 cases of ER^+^ breast cancer having clinical follow-up data, Pearson Chi-squared test showed eIF4E expression was significantly associated with Tamoxifen response (*P* = 0.016; Fig. 7d), with high eIF4E expression found in 57.1% patients with tamoxifen resistance; while low eIF4E expression was found in 67.3% tamoxifen sensitive patients (Fig. 7d). Tamoxifen resistance is defined as those patients with ER^+^ primary breast cancer who were treated with adjuvant Tamoxifen but developed subsequent metastasis or local relapse. These results confirm the positive association of high eIF4E expressing primary breast cancer with tamoxifen resistance.

## Discussion

A recent report suggested that tamoxifen resistance involves genome-wide translational reprogramming to select for the translation of mRNAs mediated by increased expression and availability of eIF4E, through phosphorylation and its increased availability by hyperactive mTOR [28]. Through a knockdown approach, they uncovered that mitogen-activated protein kinase (MAPK)-interacting serine/threonine kinase (MNK), which mediates the phosphorylation of eIF4F at Ser209, is essential for modulating the downstream pathway of eIF4E, and further identified *RUNX2* to be involved in modulating tamoxifen resistance. Re-sensitization to tamoxifen was restored by reducing eIF4E expression or mTOR activity, silencing *RUNX2 or* by blocking MNK1 phosphorylation of eIF4E [28]. Furthermore, they predicted that increased levels or availability of eIF4E and increased eIF4E S209 phosphorylation by MNK1 would promote selective increased translation of *RUNX2* mRNA.

These findings suggest that high expression of RUNX2 would be expected to be involved in modulating tamoxifen response [28]. Indeed we found that treating the tamoxifen-resistant cell line LCC2 with MNK inhibitor could reverse tamoxifen resistance (Fig. S10a) and reduce the expression of *RUNX2* (Fig. S10b). However, contrary to what was predicted [28], overexpressing *eIF4E* in MCF-7 and ZR-75 cells did not appear to alter the expression of *RUNX2* (Fig. S10c). Moreover, using the same lysates that had demonstrated reduced *RUNX2* expression, MNK inhibitor treatment only resulted in marginal reduction of *FOXM1* expression (Fig. S10d), suggesting that alteration of tamoxifen response through increased eIF4E expression may also be mediated without phosphorylation of eIF4E. To further investigate this possibility, we created two eIF4E mutants which were eIF4E S209A (nonphosphorylatable) and eIF4E S209D (phosphomimetic). The results from qPCR demonstrated that wild type as well as both the mutants exerted similar effect on mRNA (Fig. S11a) and protein levels of ERα and FOXM1 (Fig. S11b, c), indicating modulation of ERα and FOXM1 expression by eIF4E overexpression is independent of its S209 phosphorylation status.

Our study, in contrast was based on an overexpression model system for transcriptome and translatome analysis which identified alteration in the estrogen and FOXM1 signalling pathways in eIF4E overexpressing cells. The tamoxifen resistance conferred by overexpressing eIF4E in tamoxifen sensitive cells could be reversed on FOXM1 knockdown (Figs. 6a, c and S12a), rather than by blocking eIF4E phosphorylation. Indeed, treatment of MNK1 inhibitor in stably *eIF4E* overexpressing cells did not modulate the expression of *FOXM1* (Fig. S12b), nor could it reverse tamoxifen sensitivity (Fig. S12c), even with significant reduction of eIF4E phosphorylation (Fig. S12d). Taken together, these findings are supportive that the promotion of tamoxifen resistance in ER^+^ breast cancer through modulating the translation of the selective mRNAs mediated by eIF4E overexpression is independent of the phosphorylation status at S209.

Consistent with our polysome fractionation experiments which show that eIF4E modulated protein synthesis of both ERα and FOXM1, the correlation between eIF4E with ERα and FOXM1 was verified in vivo using 134 Chinese breast cancer samples. High expression of eIF4E was associated with high expression of ERα and FOXM1 by IHC staining. It was also significantly correlated with ERα and PR status retrieved from clinical records, which has been independently assessed by IHC at the time of diagnosis. However it was the upregulation of ERα which mediated further upregulation of FOXM1, through transcription. FOXM1 is an oncogenic transcription factor that controls key regulators of tumorigenesis for which we have previously demonstrated its transcription is under the control of ERα [20]. Studies have demonstrated that down regulation of FOXM1 favours apoptosis [29, 30], thus upregulation would counteract the effect of Tamoxifen on apoptosis, explaining why increased eIF4E while causing increased levels of ERα also resulted in tamoxifen resistance. Our study therefore uncovers a novel mechanism conferring tamoxifen resistance via both ERα dependent and independent pathways as eIF4E could modulate FOXM1 via translation.

Needless to say, there are other mechanisms through which *eIF4E* overexpression can mediate tamoxifen resistance. Firstly, *eIF4E* overexpression may directly promote protein synthesis of two other important estradiol targets, MYC and cyclin D1 that have eIF4E-sensitive mRNA, which are known to play major roles in driving ER^+^ breast cancer growth, though our data suggests they more important in driving cell proliferation rather than modulating tamoxifen response (Figs. S8 and S9). Secondly, as *eIF4E* overexpression promotes the expression of ERα, with the increasing amount of ERα available in cells, the original tamoxifen dosage might become insufficient to suppress the ER-signalling pathway and inhibit cancer cell growth. Thirdly, *eIF4E* overexpression might promote the PI3K-Akt pathway [31], through upregulation of NBS1 [31], or through upregulation of ERα, which in turn could activate PI3K through its non-genomic signalling pathway [32]. Activation of the PI3K/Akt pathway could lead to activation of ER-activity without oestrogen stimulation, further contributing to cell growth under tamoxifen treatment. Finally, eIF4E might confer tamoxifen resistance via oestrogen receptor independent pathway due to the activity of mTOR and MNK1 [28], one of the downstream modulators being RUNX2 [33] which is found to be important in breast tumour growth and metastasis [34, 35]. Moreover, RUNX2 could regulate the WNT/β-catenin and TCF-β signalling pathways, two pathways essential to tamoxifen resistance [36, 37]. In tamoxifen-resistant cells, these mechanisms might work together, forming a single or multiple positive feedback loops, amplifying their effects and protecting cells from induced apoptosis.

Our study which demonstrates phosphorylation independent eIF4E translational reprogramming of selective mRNAs determining tamoxifen resistance is novel. While confirming that eIF4E can govern the protein synthesis of ERα and FOXM1, in ER^+^ breast cancer, FOXM1 is a critical ERα target gene for modulating tamoxifen response. Coupled with eIF4E translational regulation, our study highlights yet another important mechanism conferring tamoxifen resistance via both ERα dependent and independent pathways.

## Materials and methods

### Cell culture

The human breast cancer cell lines MCF-7, ZR-75 and non-tumourgenic breast epithelial cell line MCF-10A, were obtained from the American Type Culture Collection (ATCC). AK-47 and LCC2 were a kind gift from Prof. Robert Clarke (Georgetown University, Washington, D.C). All cell lines were authenticated by STR profiling in 2013 [25]. All cell lines were tested to confirm no mycoplasma contamination.

### RNA sequencing

cDNA libraries were prepared by KAPA Stranded mRNA-Seq Kit (KR0960-v3.15) and the sequencing was performed using NovaSeq 6000 by Centre for Genomic Sciences (The University of Hong Kong). The data can be downloaded (https://www.ncbi.nlm.nih.gov/geo/query/acc.cgi?acc=GSE132851).

### RNA stability assay

The cells were treated with 5 µg/mL of Actinomycin D (ActD) for 0 and 24 h. The expression levels of ERα and FOXM1 mRNA were compared between the two time points. In(C_24_/C_0_) = −kt was used to determine value of k where C_24_ and C_0_ are expression levels at 24 and 0 h, k is a decay constant and t is the time.

### In silico analyses

Messenger RNA sequences of ERα (NM_000125), FOXM1 (NM_001243088.1), GAPDH (NM_002046), β-actin (NM_001101), Myc (NM_002467) and cyclin D1 (NM_053056) were used for analysis. The minimum free energies of their 5′-UTRs and the corresponding secondary structures were predicted in silico by online software named ‘RNAfold’ (http://rna.tbi.univie.ac.at/cgi-bin/RNAfold.cgi).

### Polysome fractionation experiments

Ribosomal fractions actively translating mRNA were separated from those inactively translating mRNAs through 5 to 50% sucrose gradient by ultracentrifugation. Messenger RNAs associated with three or less ribosomes were pooled together as light weight fraction (fractions 13–17) and mRNAs associated with more than three ribosomes were pooled together as heavy weight fraction (fractions 19–23). The RNA samples extracted from fractions 19 to 23 were pooled and used for translatomic study through RNA sequencing [28].

### Total RNA extraction

Total RNAs were extracted using TRIzol (Invitrogen) following manufacturer’s protocol. The concentration of RNA was measured by NanoDrop (Invitrogen).

### Reverse transcription and real-time quantitative PCR

SuperScript III reverse transcriptase (Invitrogen) was used in reverse transcription-polymerase chain reaction. ABI 7900HT Fast Real-time PCR system (Applied Biosystems) was employed in real-time quantitative PCR (RT-qPCR) reaction. The primer sequences listed in Supplementary Table 5.

### Transient transfection and siRNA knockdown

Transient transfections and siRNA knockdown were performed using lipofectamine 2000 (Invitrogen) following manufacturer’s protocol. *pCMV6_eIF4E* (#SC118908, Origene) was used to overexpress and establish the stable cell lines. All siRNAs (ON-TARGETplus) were purchased from Dharmacon.

### Cellular assays

Cell viability was measured by MTT assay as previously described [25]. Caspase-Glo^®^ 3/7 Assay (Promega) and ERE-E1b-Luciferase reporter assay were employed [38] to measure caspase activity and ERα activity respectively. Tecan200 microplate reader was used to record absorbance and luminance.

### Western blot

Cells were harvested and lysed in 1× ice-cold Cell Lysis Buffer (Cell Signaling Technology) supplemented with 1× Complete Protease Inhibitor Cocktail–EDTA free (Roche) and 1 mM PMSF. Proteins were separated by SDS-PAGE, transferred onto PVDF membrane (BioRad), and probed for overnight with primary antibodies at 4 °C. The membrane was incubated with secondary antibodies including: conjugated horseradish peroxidase (HRP) anti-mouse (DAKO, 1:5000) and HRP anti-rabbit (DAKO, 1:2000). The membrane was exposed to X-ray film (Fujifilm) and the film was developed in automatic x-ray film processor.

### Human breast cancer samples, immunohistochemistry and tissue microarray analysis

Cases diagnosed with breast cancer between the years 1992 to 2001 were retrieved from the records of the Department of Pathology, Queen Mary Hospital, Hong Kong, with approval by the Institutional Review Board of the University of Hong Kong (UW 08-147). Five to ten year clinical follow-up data and clinic-pathological information were available for analysis. Cases defined as tamoxifen resistant were patients whose primary breast cancer was positive for ERα, were treated with tamoxifen after surgery, and who subsequently developed local recurrence or distant metastases. Histological sections of all cases were reviewed by the pathologist and the selected areas were marked for construction of tissue microarray (TMA) blocks.

The immunohistochemistry (IHC) staining was performed as previously described [24]. The staining intensities and percentages of each protein in the cytoplasm or in the nucleus were scored by two independent individuals in a semi-quantitative fashion as previously described and the average taken [24].

### Data analysis

Sequencing reads were first filtered for adapter sequence and low quality sequence followed by retaining only reads with read length ≥40 bp. Subsequently, sequencing reads were filtered for rRNA sequence and remaining reads were used for downstream analysis. Reads were mapped to the reference genome (Human Genome GRCh38) using STAR (Version 2.5.2) with default parameters [39]. Expression analysis was done using EBSeq (Version 1.18) [40]. The expression was quantified by RSEM (Version 1.2.31) [41]. Heatmap showing the differentially expressed genes was created by CIMminer (National Cancer Institute). Pathway enrichment analysis was performed using Kyoto Encyclopedia of Genes and Genomes database (KEGG). The analysis was performed on ConsensusPathDB-human performed [42]. A pathway contained at least three genes.

Results were shown as mean ± s.d. from three independent experiments. The mean values of the experimental group and the control group were compared by independent samples students’ *t*-test in Excel (Microsoft). Two way ANOVA was used for experiments with more than two groups. IBM SPSS Statistics 20 was used in statistical analysis of TMA data. Expression levels of eIF4E in tumour and non-tumour were compared by Mann–Whitney test. Correlations were assessed by Pearson’s chi-square test. The results were considered as statistically significant when the *P* < 0.05.

## Supplementary information

Supplementary table 1

Supplementary table 2

Supplementary table 3

Supplementary table 4

Supplementary table 5

Supplementary table legends

Supplementary figure 1

Supplementary figure 2

Supplementary figure 3

Supplementary figure 4

Supplementary figure 5

Supplementary figure 6

Supplementary figure 7

Supplementary figure 8

Supplementary figure 9

Supplementary figure 10

Supplementary figure 11

Supplementary figure 12

Supplementary figure legend

Supplementary material and method
