# Supplementary material for: Phosphorylation independent eIF4E translational reprogramming of selective mRNAs determines tamoxifen resistance in breast cancer
Source: Oncogene. 2020 Feb 17;39(15):3206–17. doi: 10.1038/s41388-020-1210-y (PMC7142019; doi:10.1038/s41388-020-1210-y)
Supplement: Supplementary file 6 — Supplementary table legends [file 41388_2020_1210_MOESM6_ESM.docx]

**Supplementary table legends**

**Supplementary table 1 The effect of eIF4E overexpression on total mRNA expression.**

**Supplementary table 2 The effect of eIF4E overexpression on mRNA in polysome fractions.**

**Supplementary table 3 KEGG pathway enrichment analysis on eIF4E overexpression on transcriptome.**

**Supplementary table 4 KEGG pathway enrichment analysis on eIF4E overexpresion on translatome.**

**Supplementary table 5 Primer sequences used in this study (5'-->3').**
