# Supplementary material for: Phosphorylation independent eIF4E translational reprogramming of selective mRNAs determines tamoxifen resistance in breast cancer
Source: Oncogene. 2020 Feb 17;39(15):3206–17. doi: 10.1038/s41388-020-1210-y (PMC7142019; doi:10.1038/s41388-020-1210-y)
Supplement: Supplementary file 7 — Supplementary figure 1 [file 41388_2020_1210_MOESM7_ESM.pptx]

## Slide 1
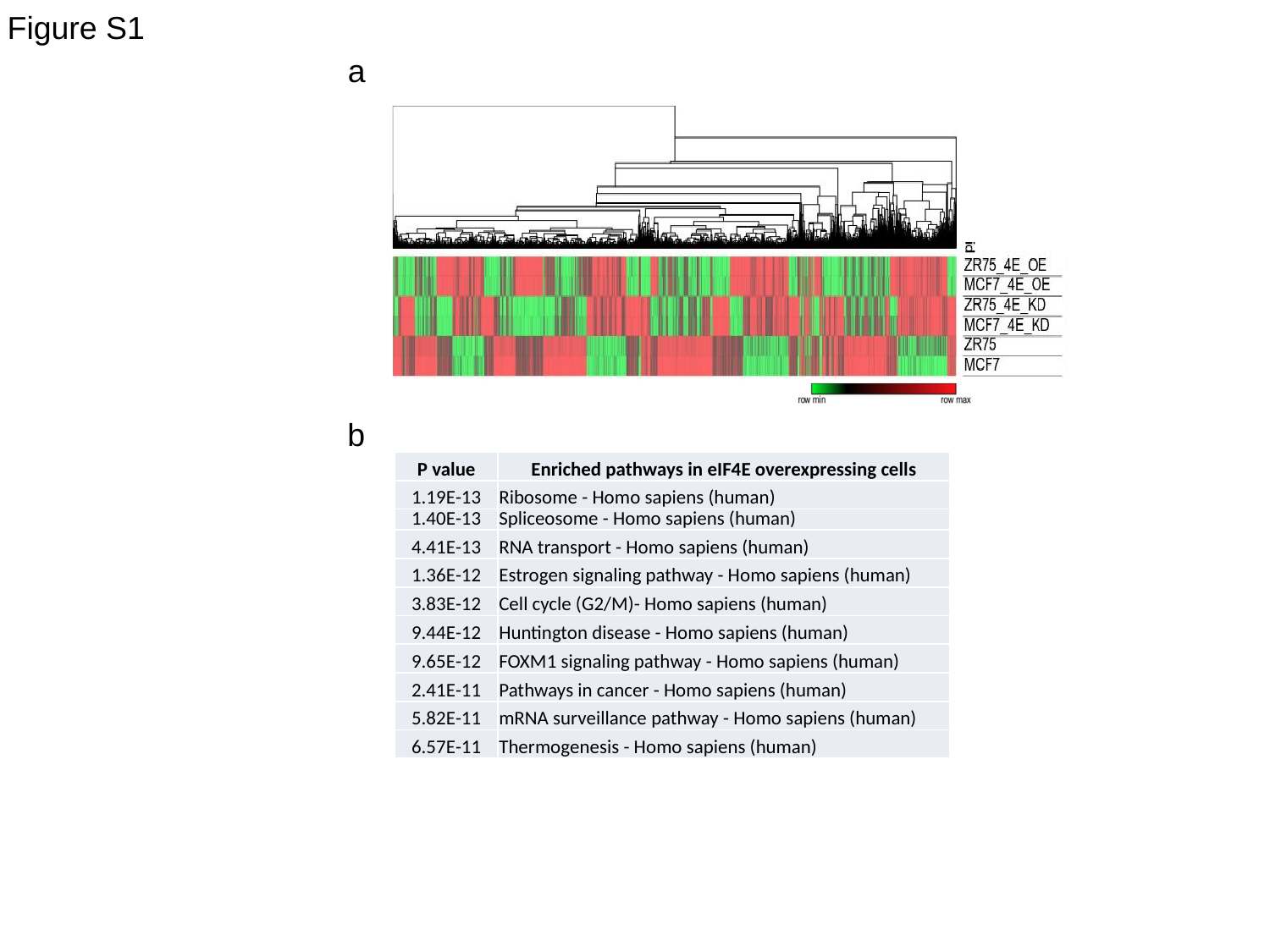

Figure S1
a
b
| P value | Enriched pathways in eIF4E overexpressing cells |
| --- | --- |
| 1.19E-13 | Ribosome - Homo sapiens (human) |
| 1.40E-13 | Spliceosome - Homo sapiens (human) |
| 4.41E-13 | RNA transport - Homo sapiens (human) |
| 1.36E-12 | Estrogen signaling pathway - Homo sapiens (human) |
| 3.83E-12 | Cell cycle (G2/M)- Homo sapiens (human) |
| 9.44E-12 | Huntington disease - Homo sapiens (human) |
| 9.65E-12 | FOXM1 signaling pathway - Homo sapiens (human) |
| 2.41E-11 | Pathways in cancer - Homo sapiens (human) |
| 5.82E-11 | mRNA surveillance pathway - Homo sapiens (human) |
| 6.57E-11 | Thermogenesis - Homo sapiens (human) |
