# Supplementary material for: Phosphorylation independent eIF4E translational reprogramming of selective mRNAs determines tamoxifen resistance in breast cancer
Source: Oncogene. 2020 Feb 17;39(15):3206–17. doi: 10.1038/s41388-020-1210-y (PMC7142019; doi:10.1038/s41388-020-1210-y)
Supplement: Supplementary file 8 — Supplementary figure 2 [file 41388_2020_1210_MOESM8_ESM.pptx]

## Slide 1
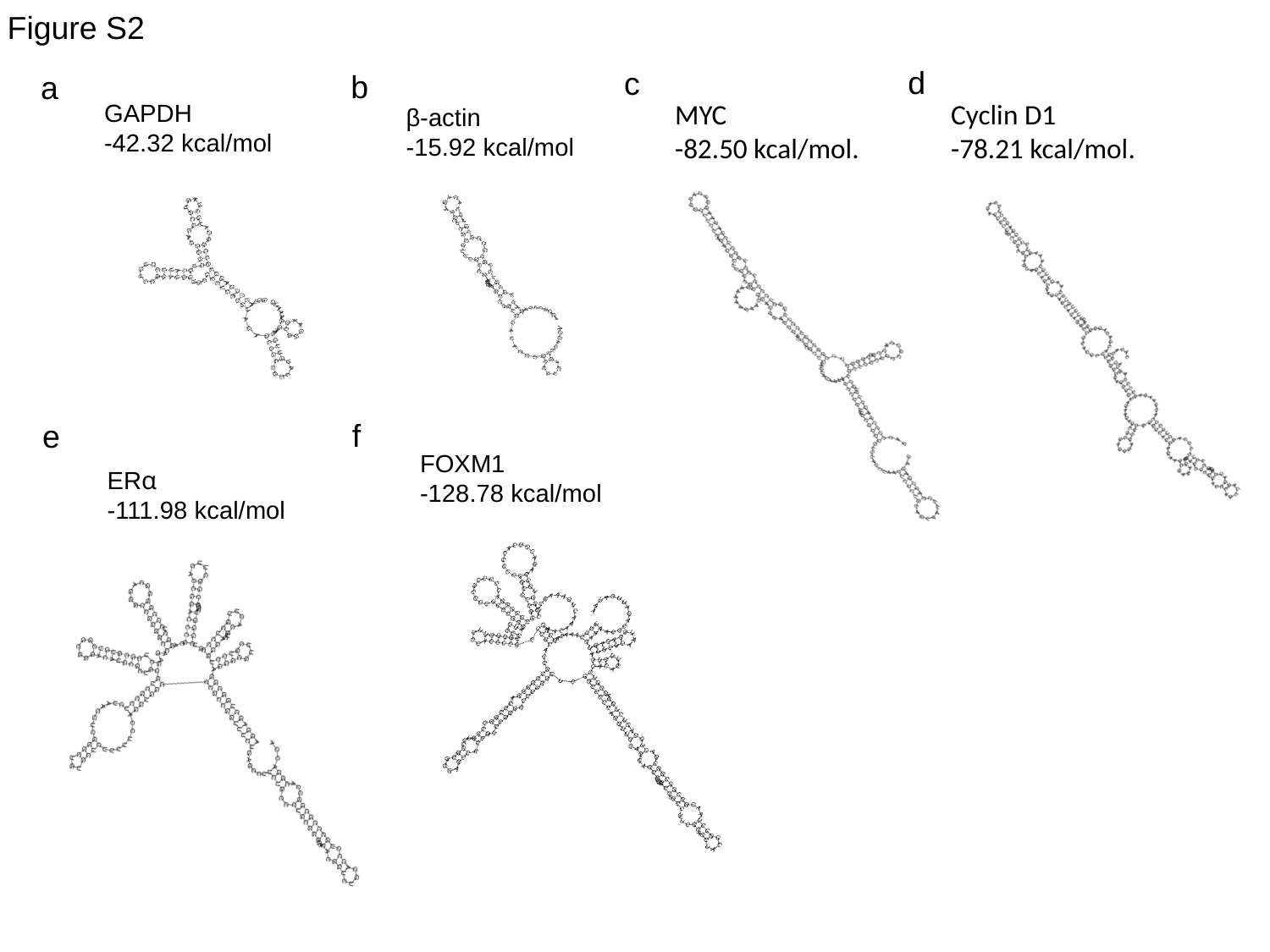

Figure S2
d
c
b
a
MYC
-82.50 kcal/mol.
Cyclin D1
-78.21 kcal/mol.
GAPDH
-42.32 kcal/mol
β-actin
-15.92 kcal/mol
f
e
FOXM1
-128.78 kcal/mol
ERα
-111.98 kcal/mol
