# Supplementary material for: Phosphorylation independent eIF4E translational reprogramming of selective mRNAs determines tamoxifen resistance in breast cancer
Source: Oncogene. 2020 Feb 17;39(15):3206–17. doi: 10.1038/s41388-020-1210-y (PMC7142019; doi:10.1038/s41388-020-1210-y)
Supplement: Supplementary file 10 — Supplementary figure 4 [file 41388_2020_1210_MOESM10_ESM.pptx]

## Slide 1
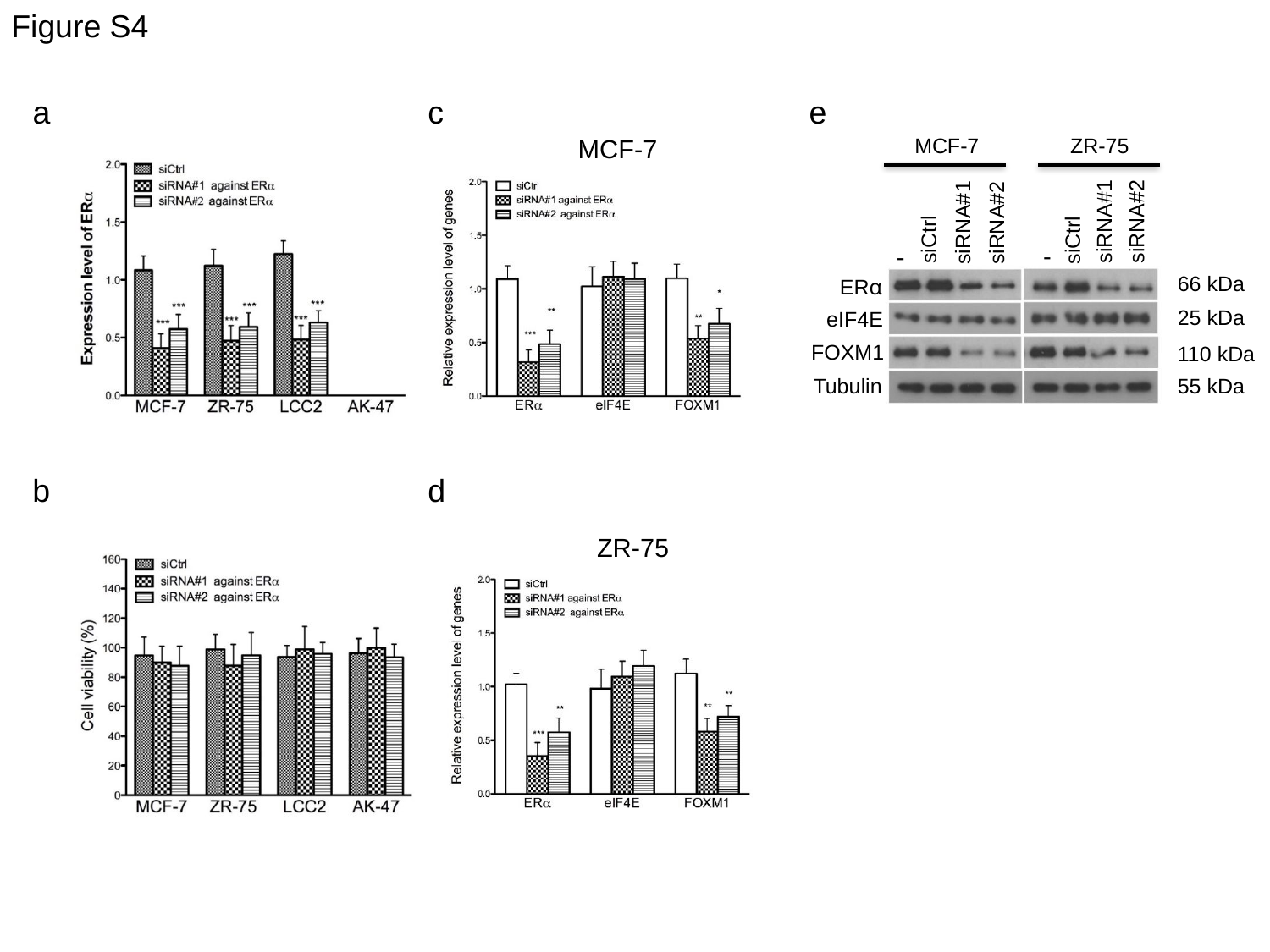

Figure S4
a
c
e
MCF-7
MCF-7
ZR-75
siRNA#1
siRNA#2
siRNA#1
siRNA#2
siCtrl
siCtrl
-
-
66 kDa
ERα
25 kDa
eIF4E
FOXM1
110 kDa
55 kDa
Tubulin
b
d
ZR-75
