# Supplementary material for: Phosphorylation independent eIF4E translational reprogramming of selective mRNAs determines tamoxifen resistance in breast cancer
Source: Oncogene. 2020 Feb 17;39(15):3206–17. doi: 10.1038/s41388-020-1210-y (PMC7142019; doi:10.1038/s41388-020-1210-y)
Supplement: Supplementary file 13 — Supplementary figure 7 [file 41388_2020_1210_MOESM13_ESM.pptx]

## Slide 1
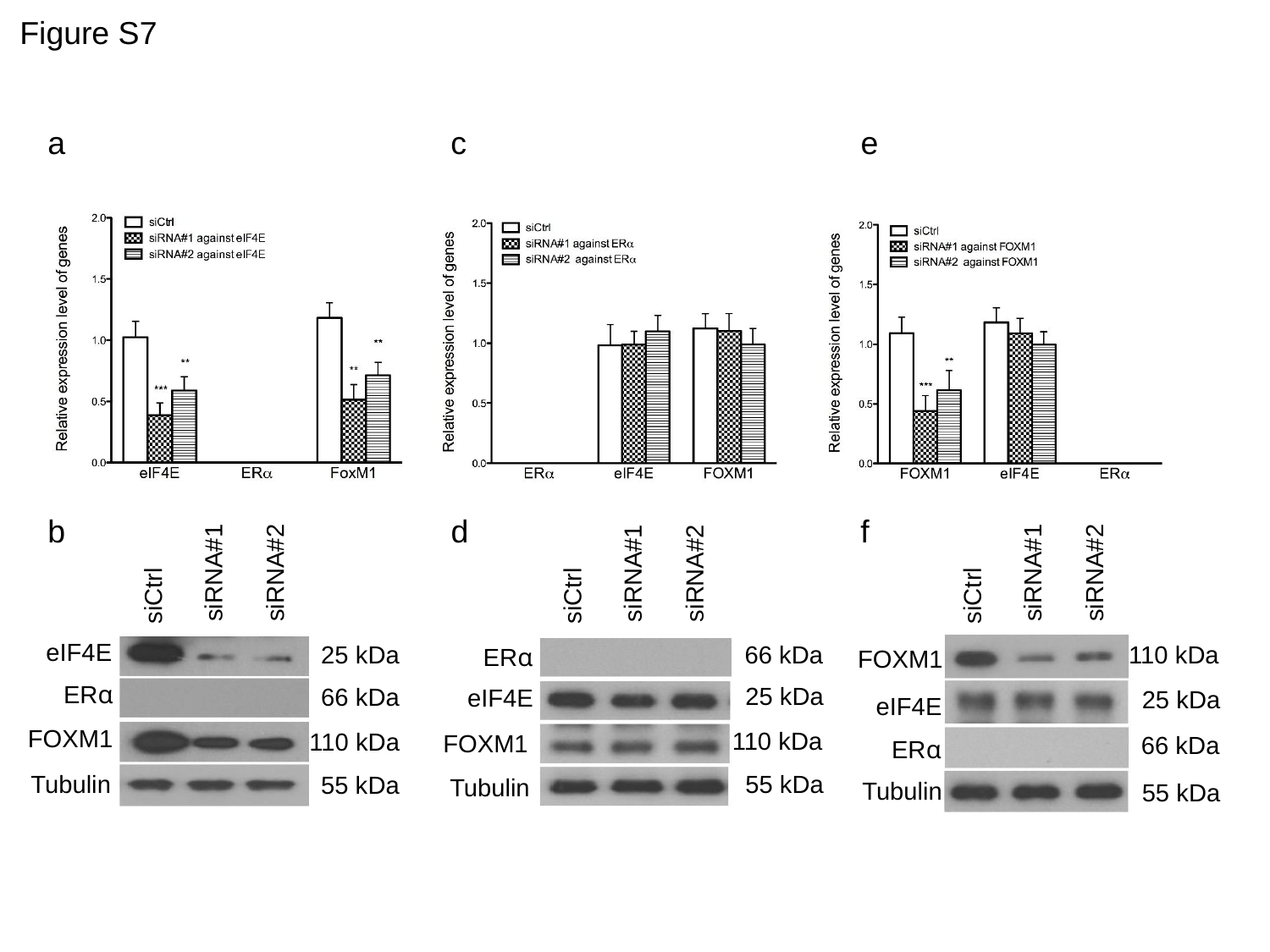

Figure S7
a
c
e
b
d
f
siRNA#1
siRNA#2
siCtrl
110 kDa
FOXM1
25 kDa
eIF4E
66 kDa
ERα
Tubulin
55 kDa
siRNA#1
siRNA#2
siCtrl
eIF4E
25 kDa
ERα
66 kDa
FOXM1
110 kDa
Tubulin
55 kDa
siRNA#1
siRNA#2
siCtrl
66 kDa
ERα
25 kDa
eIF4E
110 kDa
FOXM1
55 kDa
Tubulin
