# Supplementary figures and images for: Phosphorylation independent eIF4E translational reprogramming of selective mRNAs determines tamoxifen resistance in breast cancer
Source: Oncogene. 2020 Feb 17;39(15):3206–17. doi: 10.1038/s41388-020-1210-y (PMC7142019; doi:10.1038/s41388-020-1210-y)

## Slide 1
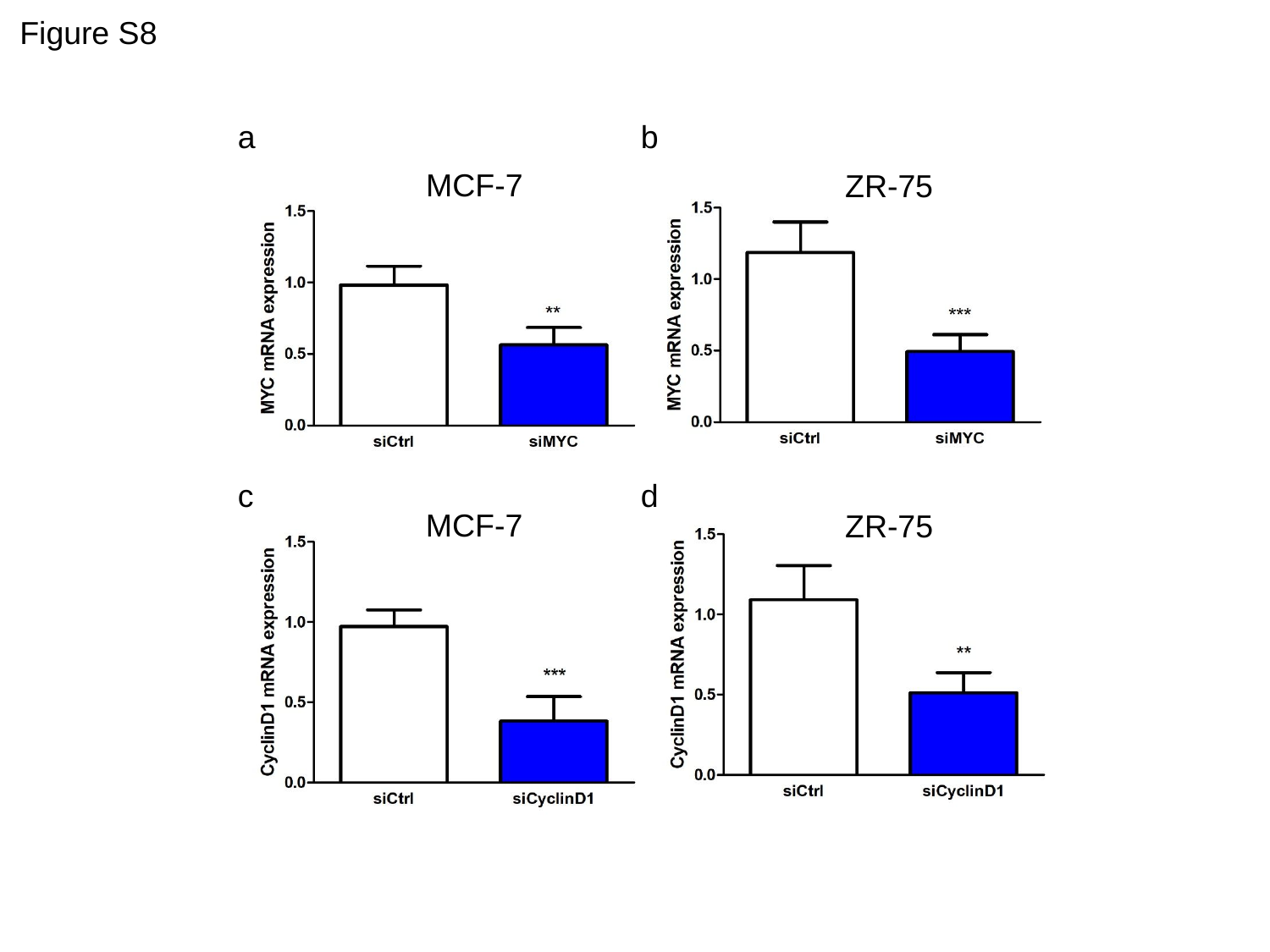

Figure S8
a
b
MCF-7
ZR-75
c
d
MCF-7
ZR-75

Supplement: Supplementary file 14 — Supplementary figure 8 [file 41388_2020_1210_MOESM14_ESM.pptx]

## Slide 1
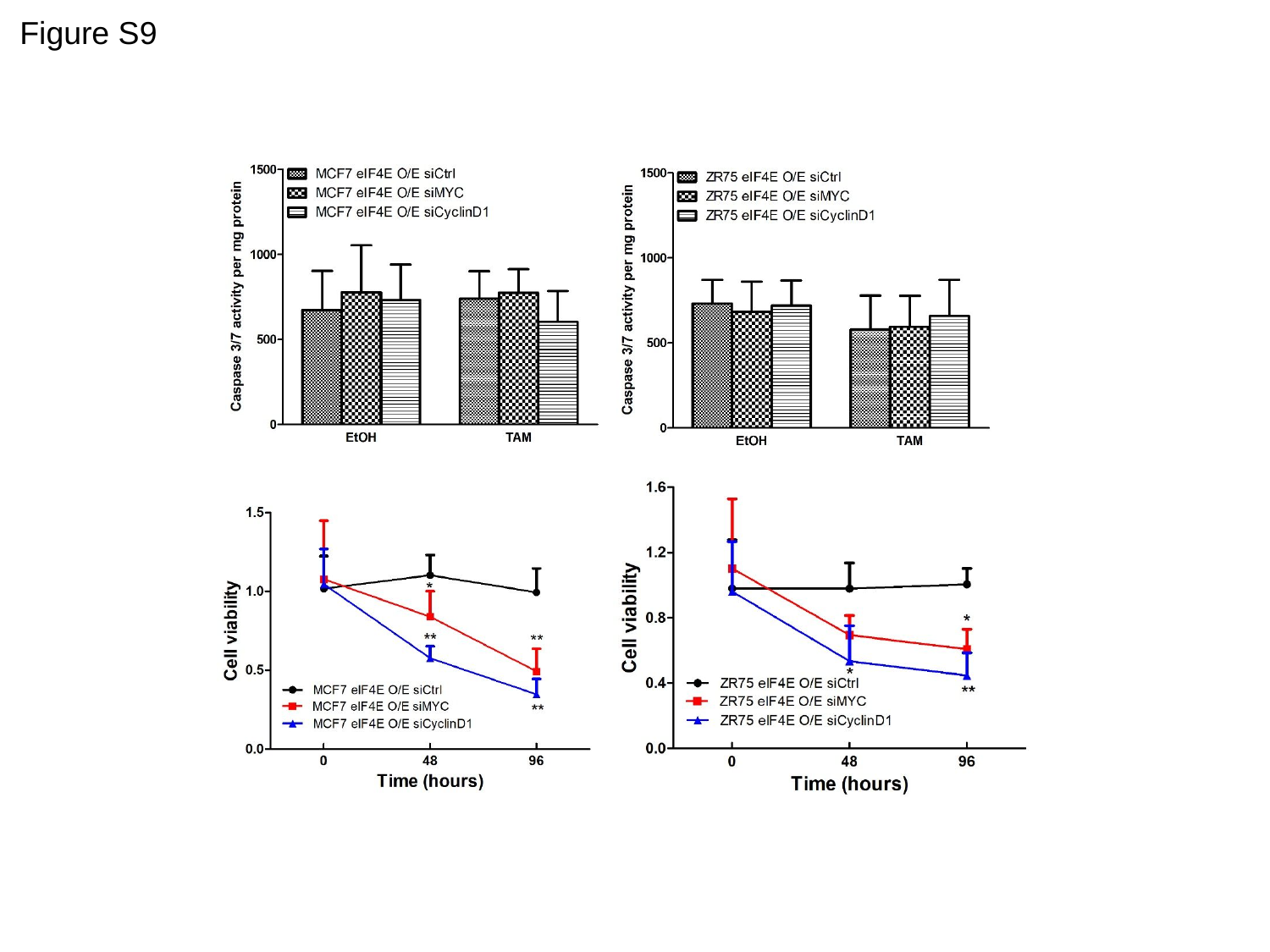

Figure S9

Supplement: Supplementary file 15 — Supplementary figure 9 [file 41388_2020_1210_MOESM15_ESM.pptx]

## Slide 1
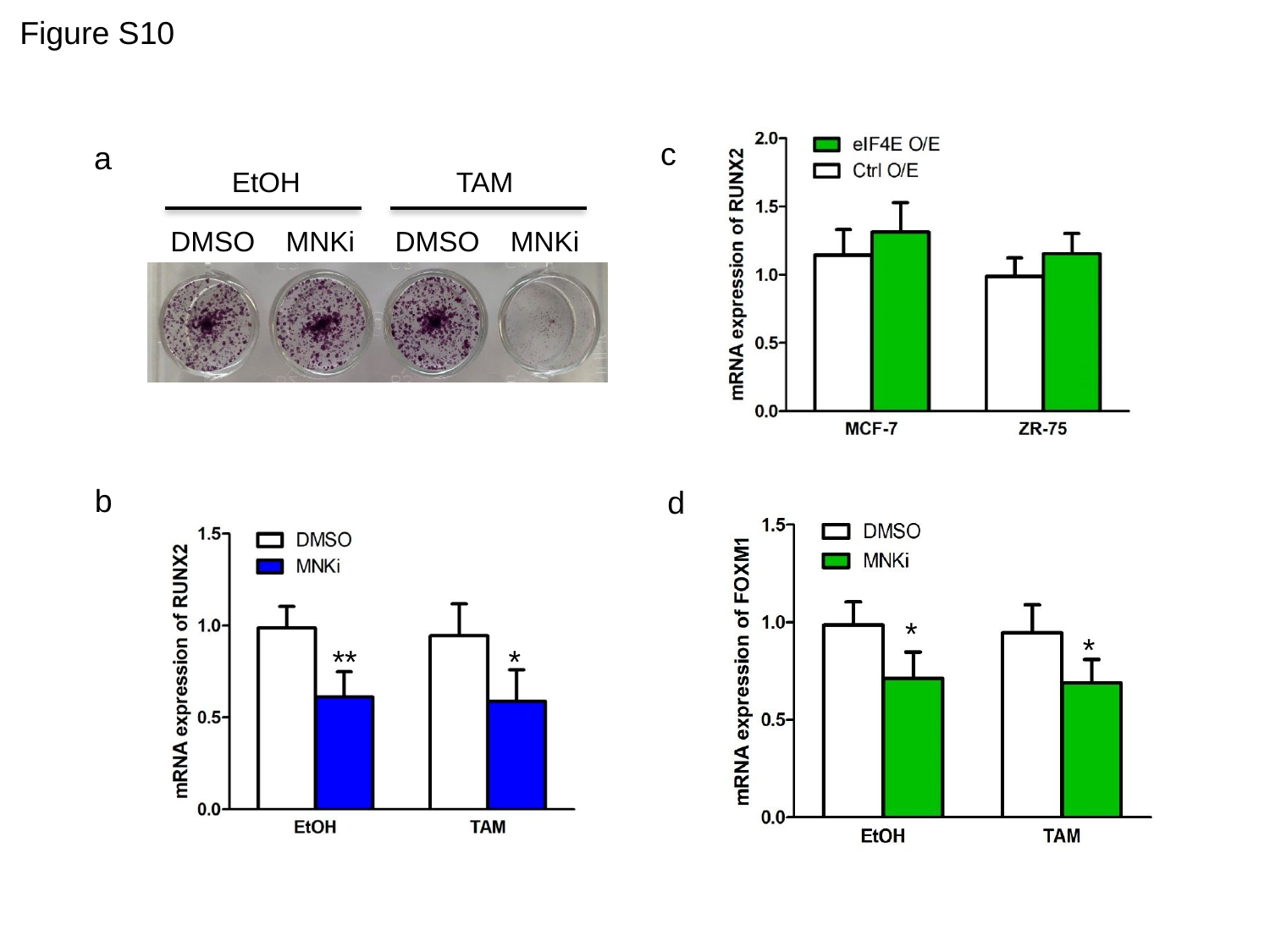

Figure S10
c
a
TAM
EtOH
MNKi
DMSO
MNKi
DMSO
b
d
*
*
**
*

Supplement: Supplementary file 16 — Supplementary figure 10 [file 41388_2020_1210_MOESM16_ESM.pptx]
