# Supplementary material for: Phosphorylation independent eIF4E translational reprogramming of selective mRNAs determines tamoxifen resistance in breast cancer
Source: Oncogene. 2020 Feb 17;39(15):3206–17. doi: 10.1038/s41388-020-1210-y (PMC7142019; doi:10.1038/s41388-020-1210-y)
Supplement: Supplementary file 17 — Supplementary figure 11 [file 41388_2020_1210_MOESM17_ESM.pptx]

## Slide 1
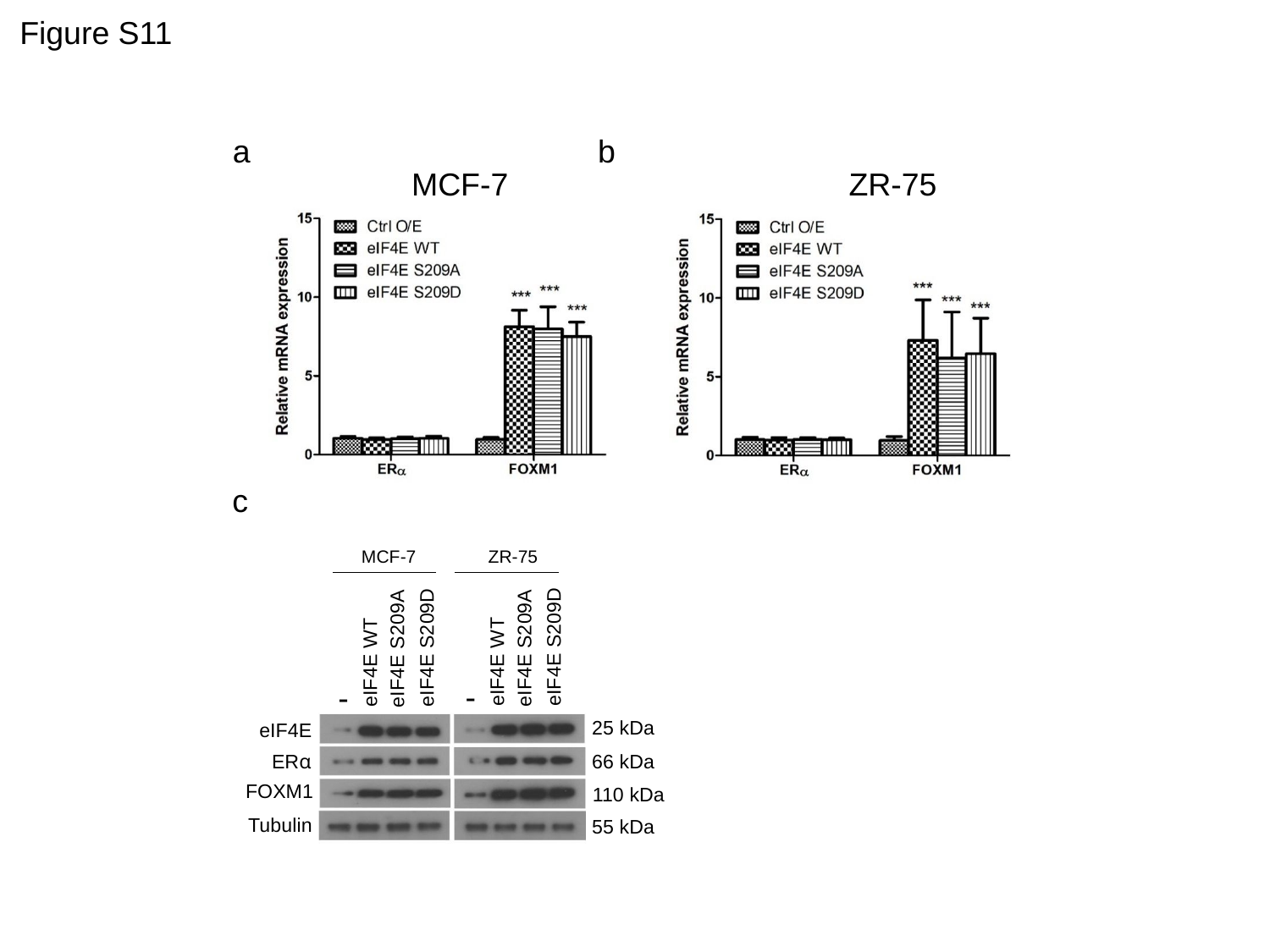

Figure S11
a
b
MCF-7
ZR-75
c
MCF-7
ZR-75
eIF4E S209D
eIF4E S209D
eIF4E S209A
eIF4E S209A
eIF4E WT
eIF4E WT
-
-
25 kDa
eIF4E
ERα
66 kDa
FOXM1
110 kDa
Tubulin
55 kDa
