# Supplementary material for: Phosphorylation independent eIF4E translational reprogramming of selective mRNAs determines tamoxifen resistance in breast cancer
Source: Oncogene. 2020 Feb 17;39(15):3206–17. doi: 10.1038/s41388-020-1210-y (PMC7142019; doi:10.1038/s41388-020-1210-y)
Supplement: Supplementary file 18 — Supplementary figure 12 [file 41388_2020_1210_MOESM18_ESM.pptx]

## Slide 1
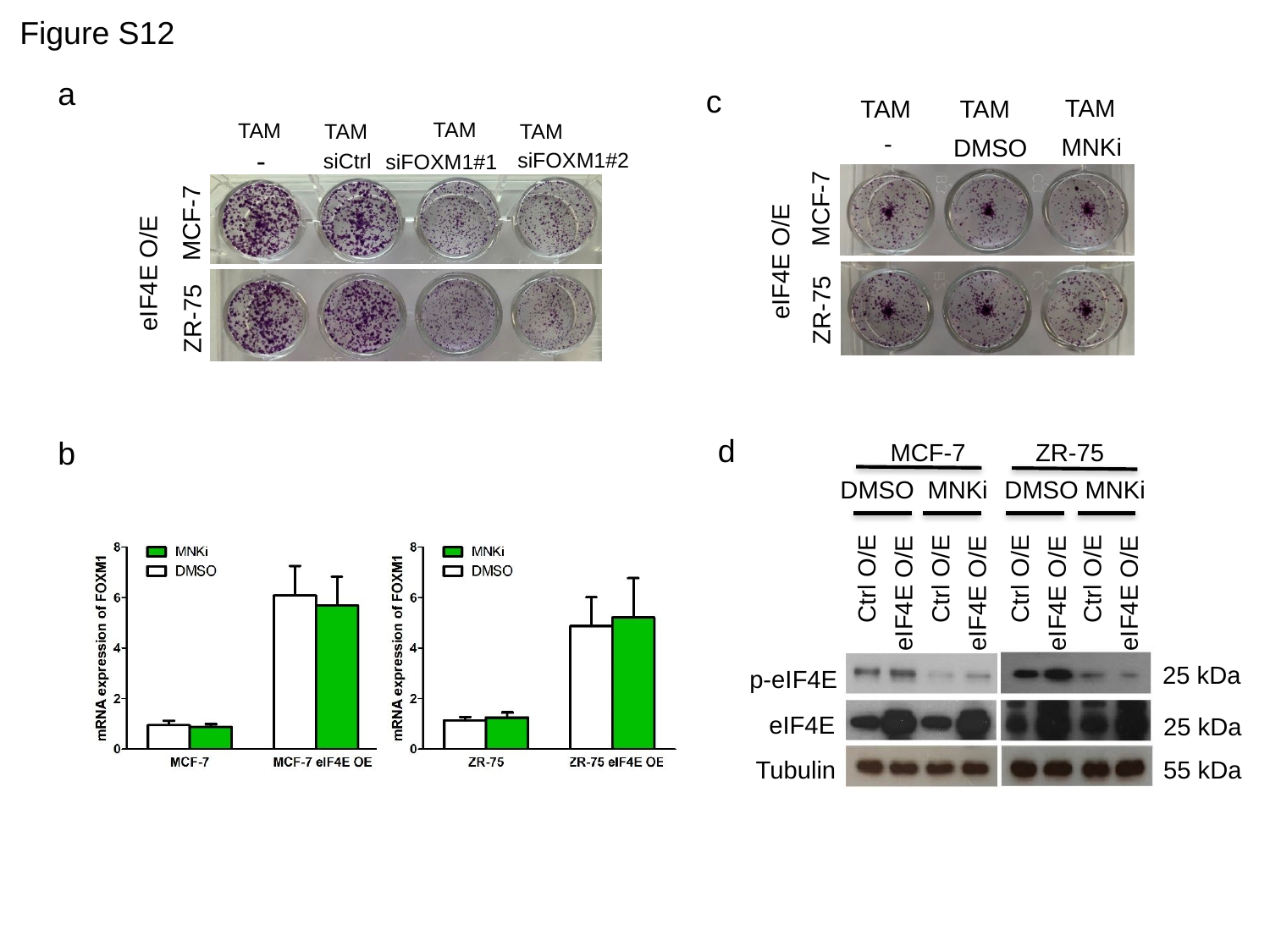

Figure S12
a
c
TAM
TAM
TAM
-
MNKi
DMSO
MCF-7
eIF4E O/E
ZR-75
TAM
TAM
TAM
TAM
-
siFOXM1#2
siCtrl
siFOXM1#1
MCF-7
eIF4E O/E
ZR-75
d
b
MCF-7
ZR-75
DMSO
MNKi
DMSO
MNKi
Ctrl O/E
Ctrl O/E
Ctrl O/E
Ctrl O/E
eIF4E O/E
eIF4E O/E
eIF4E O/E
eIF4E O/E
25 kDa
p-eIF4E
eIF4E
25 kDa
55 kDa
Tubulin
