# Supplementary material for: Phosphorylation independent eIF4E translational reprogramming of selective mRNAs determines tamoxifen resistance in breast cancer
Source: Oncogene. 2020 Feb 17;39(15):3206–17. doi: 10.1038/s41388-020-1210-y (PMC7142019; doi:10.1038/s41388-020-1210-y)
Supplement: Supplementary file 19 — Supplementary figure legend [file 41388_2020_1210_MOESM19_ESM.docx]

**Supplementary figure legends**

**Figure S1 Modulation of *eIF4E* expression in MCF-7 and ZR-75 breast cancer cells could induce distinctive molecular alterations.** a) Heatmap showed the gene expression profiles. The cells were treated with either *pCMV6_eIF4E* or eIF4E siRNA#1 for 72 hours. RNA sequencing was performed to determine the effect of eIF4E alteration on gene expressions. Heatmap was used to show the profiles of the molecular features. 4E_OE represents eIF4E overexpression. 4E_KD represents eIF4E knockdown. b) Top 10 molecular pathways being enriched by overexpression of eIF4E. KEGG pathway analysis was performed to identify pathways which were potentially enriched in eIF4E overexpressing breast cancer cells.

**Figure S2 Secondary structures in 5’-UTR of a) GAPDH, b) β-actin, c) MYC, d) Cyclin D1, e) ERα and f) FOXM1.**

The structures were predicted by RNAFold. The minimum free energy was indicated.

**Figure S3 The effect of eIF4E knockdown on mRNA and protein expression of ERα and FOXM1.**

a) The knockdown efficiency of the siRNAs against *eIF4E*. Knockdown of *eIF4E* was mediated by transfection of 20 pmol of siRNA#1 or siRNA#2. 20 pmol of non-targeting siRNA (siCtrl) was used as the control. qPCR was determined after 72 hours posttransfection. b) The effect of eIF4E knockdown on cell viability after 72 hours posttransfection was determined by MTT assay. The effect of *eIF4E* knockdown on mRNA levels of *ERα* and *FOXM1* was determined in c) MCF-7 and d) ZR-75 cells. qPCR was determined after 72 hours posttransfection. e) The effect of *eIF4E* knockdown on protein levels of ERα and FOXM1 was determined in MCF-7 and ZR-75 cells. Western blot was performed with tubulin as the loading control. Untransfected control was used as the reference for the normalization in qPCR. Actin was used as the internal control for qPCR. Results were expressed as mean ± s.d. from three independent experiments. *, ** and *** indicate a statistical significance with *P*<0.05, *P*<0.01 and *P*<0.001 by independent student t-test respectively.

**Figure S4 The effect of *ERα* knockdown on mRNA and protein expression of eIF4E and FOXM1.**

a) The knockdown efficiency of the siRNAs against *ERα*. Knockdown of *ERα* was mediated by transfection of 50 pmol of siRNA#1 or siRNA#2. 50 pmol of non-targeting siRNA (siCtrl) was used as the control. qPCR was determined after 72 hours posttransfection. b) The effect of *ERα* knockdown on cell viability after 72 hours posttransfection was determined by MTT assay. The effect of *ERα* knockdown on mRNA levels of *eIF4E* and *FOXM1* was determined in c) MCF-7 and d) ZR-75 cells. qPCR was determined after 72 hours posttransfection. e) The effect of *ERα* knockdown on protein levels of eIF4E and FOXM1 was determined in MCF-7 and ZR-75 cells. Western blot was used to determine the protein expression. Tubulin was used as the loading control. Untransfected control was used as the reference for the normalization in qPCR. Actin was used as the internal control for qPCR. Results were expressed as mean ± s.d. from three independent experiments. *, ** and *** indicate a statistical significance with *P*<0.05, *P*<0.01 and *P*<0.001 by independent student t-test respectively.

**Figure S5 The effect of *FOXM1* knockdown on mRNA and protein expression of eIF4E and ERα.**

a) The knockdown efficiency of the siRNAs against *FOXM1*. Knockdown of *FOXM1* was mediated by transfection of 50 pmol of siRNA#1 or siRNA#2. 50 pmol of non-targeting siRNA (siCtrl) was used as the control. qPCR was determined after 72 hours posttransfection. b) The effect of *FOXM1* knockdown on cell viability after 72 hours posttransfection was determined by MTT assay. The effect of *FOXM1* knockdown on mRNA levels of *eIF4E* and *ERα* was determined in c) MCF-7 and d) ZR-75 cells. qPCR was determined after 72 hours posttransfection. e) The effect of *FOXM1* knockdown on protein levels of eIF4E and ERα was determined in MCF-7 and ZR-75 cells. Western blot was used to determine the protein expression. Tubulin was used as the loading control. Untransfected control was used as the reference for the normalization in qPCR. Actin was used as the internal control for qPCR. Results were expressed as mean ± s.d. from three independent experiments. *, ** and *** indicate a statistical significance with *P*<0.05, *P*<0.01 and *P*<0.001 by independent student t-test respectively.

**Figure S6 The effect of modulating the expression of eIF4E, ERα or FOXM1 in turn on mRNA and protein expression of other two genes in LCC2.**

Knockdown of *eIF4E* modulated a) the mRNA levels of FOXM1 and b) the protein levels of ERα and FOXM1. Knockdown of *eIF4E* was mediated by transfection of 20 pmol of the corresponding siRNA#1 or siRNA#2. Knockdown of *ERα* modulated c) the mRNA levels and d) the protein levels of FOXM1 only. Knockdown of *ERα* was mediated by transfection of 50 pmol of the corresponding siRNA#1 or siRNA#2. Knockdown of *FOXM1* did not affect e) the mRNA levels nor f) the protein levels of eIF4E and ERα. Knockdown of *FOXM1* was mediated by transfection of 50 pmol of the corresponding siRNA#1 or siRNA#2. 20 pmol or 50 pmol of non-targeting siRNA (siCtrl) was used. qPCR was used to determine the expression of indicated candidates after 72 hours posttransfection. Actin was used as the internal control. Data was shown as mean ± s.d. from three independent experiments. Expression levels in targeting siRNA was compared to non-targeting siRNA treated group. *, **, *** indicate a statistical significance with *P*<0.05, *P*<0.01 and *P*<0.001 respectively by student t-test. Western blot was employed to determine the expression of the target proteins. Tubulin was used as the loading control.

**Figure S7 The effect of modulating the expression of eIF4E or FOXM1 in turn on mRNA and protein expression of other gene in AK-47.**

AK-47 is an ER negative cell line, hence knockdown of *ERα* was not done. Knockdown of *eIF4E* modulated a) the mRNA levels and b) the protein levels of FOXM1. Knockdown of *eIF4E* was mediated by transfection of 20 pmol of the corresponding siRNA#1 or siRNA#2. Knockdown of *FOXM1* did not affect c) the mRNA and d) the protein levels of eIF4E and ERα. Knockdown of *FOXM1* was mediated by transfection of 50 pmol of the corresponding siRNA#1 or siRNA#2. 20 pmol or 50 pmol of non-targeting siRNA (siCtrl) was used. qPCR was used to determine the expression of indicated candidates after 72 hours posttransfection. Actin was used as the internal control. Data was shown as mean ± s.d. from three independent experiments. Expression levels in targeting siRNA was compared to non-targeting siRNA treated group. *, **, *** indicate a statistical significance with *P*<0.05, *P*<0.01 and *P*<0.001 respectively by student t-test. Western blot was employed to determine the expression of the target proteins. Tubulin was used as the loading control.

**Figure S8 Knockdown efficiency of *MYC* in a) MCF-7 and b) ZR-75 and that of *Cyclin D1* in c) MCF-7 and d) ZR-75.** The cells were treated with 100 pmol of the corresponding siRNA. Total RNA was harvested 48 hours posttransfection. qPCR was employed to determine the mRNA expression of the candidates. Data shown as mean ± s.d. from three independent experiments. **, *** indicate a statistical significance with *P*<0.01 and *P*<0.001 respectively by student t-test.

**Figure S9 The effect of *MYC* and *Cyclin D1* knockdown on caspase activity and cell viability.** Knockdown of *MYC* and *Cyclin D1* did not resume the effect of tamoxifen on the activation of caspases in eIF4E overexpressing a) MCF-7 and b) ZR-75 cells. 100 pmol of the corresponding siRNA was used. 4 µM of TAM was used to treat the cells for 72 hours. Knockdown of *MYC* and *Cyclin D1* could reduce cell proliferation in eIF4E overexpressing c) MCF-7 and d) ZR-75 cells. Results shown as mean ± s.d. from three independent experiments. *, ** indicate a statistical significance with *P*<0.05 and *P*<0.01 respectively by student t-test.

**Figure S10 The effect of MNK inhibitor on LCC2.** a) Treatment of MNK inhibitor could reverse tamoxifen resistance in LCC2. Clonogenic assay was performed. The cells were treated with either EtOH or 4 µM of tamoxifen for 14 days. 10 µM of MNK inhibitor (MNKi) CGP57380 was used. b) MNK inhibitor could reduce the expression of RUNX2 in LCC2 cells. c) Overexpression of *eIF4E* did not affect the expression of RUNX2 in MCF-7 and ZR-75. 0.5 μg of *pCMV6* was used as control (Ctrl O/E) while overexpression of *eIF4E* (eIF4E O/E) was mediated by transfection of 0.5 μg of *pCMV6_eIF4E*. qPCR was used to determine the expression of *RUNX2* after 72 hours of the treatment. d) Treatment of MNK inhibitor resulted in only marginal reduced expression of *FOXM1* in LCC2 cells. 10 µM of MNK inhibitor (MNKi) CGP57380 was used. qPCR was used to determine the expression of *FOXM1* after 72 hours of the treatment. Actin was used as the internal control. Data was shown as mean ± s.d. from three independent experiments. *, ** indicate a statistical significance with *P*<0.05 and *P*<0.01 respectively by student t-test.

**Figure S11** **The effect of eIF4E overexpression on the expression of ERα and FOXM1 was independent on the phosphorylation status of S209 on eIF4E.** The effect of eIF4E S209A and eIF4E S209D on the mRNA level of ERα and FOXM1 was determined in a) MCF-7 and b) ZR-75 cells. qPCR was employed. Actin was used as the internal control. Results were shown as mean ± s.d. from three independent experiments. *** represents *P* < 0.001. c) The effect of eIF4E S209A and eIF4E S209D on the protein level of ERα and FOXM1 on MCF-7 and ZR-75 cells was determined. The cells were transfected with 2 µg of *pcDNA3.1-eIF4E WT*, *pcDNA3.1-eIF4E S209A* and *pcDNA3.1-eIF4E S209D*. Cells were harvested 48 hours post transfection. Western blot was employed. Tubulin was used as the loading control.

**Figure S12** **Tamoxifen resistance conferred by eIF4E overexpression was FOXM1 dependent.** a) The effect of FOXM1 knockdown on tamoxifen response in the eIF4E overexpressing cells. The cells were treated with 4 µM of tamoxifen. Knockdown of *FOXM1* was mediated by transfection of 50 pmol of the corresponding siRNA#1 or siRNA#2. Clonogenic assay was performed. b) The effect of MNK inhibitor on FOXM1 expression in the eIF4E overexpressing cells. qPCR was used to determine the expression of RUNX2 after 72 hours posttransfection. Actin was used as the internal control. c) The treatment of MNK inhibitor did not alter the tamoxifen response in the eIF4E overexpressing cells. The cells were treated with either EtOH or 4 µM of tamoxifen. 10 µM of MNK inhibitor (MNKi) CGP57380 was used. Clonogenic assay was performed. d) The effect of MNK inhibitor on the degree of eIF4E phosphorylation in the eIF4E overexpressing cells after 48 hours treatment. 0.5 μg of *pCMV6* was used as control (Ctrl O/E) while overexpression of *eIF4E* (eIF4E O/E) was mediated by transfection of 0.5 μg of *pCMV6_eIF4E*. Western blot was performed. Tubulin was used as the loading control. Data was shown as mean ± s.d. from three independent experiments. * indicates a statistical significance with *P*<0.05 respectively by student t-test.
