# Supplementary material for: Phosphorylation independent eIF4E translational reprogramming of selective mRNAs determines tamoxifen resistance in breast cancer
Source: Oncogene. 2020 Feb 17;39(15):3206–17. doi: 10.1038/s41388-020-1210-y (PMC7142019; doi:10.1038/s41388-020-1210-y)
Supplement: Supplementary file 20 — Supplementary material and method [file 41388_2020_1210_MOESM20_ESM.docx]

***Supplementary information***

***Supplementary materials and methods***

***Cell culture***

MCF-7 and LCC2 were cultured in DMEM containing 10% (v/v) FBS and 1% (v/v) antibiotics (penicillin, 100 U/ml; streptomycin sulfate, 100 ug/ml). MCF10-A was cultured in MEGM (ATCC) with 100 ng/ml cholera toxin. ZR-75 was cultured in phenol-free modified IEME supplemented with 10% (v/v) FBS and 1% (v/v) PS. All cells were maintained in 37^0^C humidified atmosphere supplemented with 5% CO2.

***RNA sequencing***

One microgram of total RNA was used for polyA tail RNA isolation. The purified mRNA was fragmented to 200–300 bp by incubating at 94^o^C for 6 minutes in the presence of magnesium ions. The fragmented mRNA was then applied as template to synthesize the first-strand cDNA by using random hexamer-primer and reverse transcriptase. In the second strand cDNA synthesis, the mRNA template was removed and a replacement strand was generated to form the blunt-end double-stranded (ds) cDNA. The ds cDNA underwent, 3’ adenylation and indexed adaptor ligation (xGen® Dual Index UMI Adapters). The adaptor-ligated libraries were enriched by 10 cycles of polymerase chain reaction (PCR). The libraries were denatured and diluted to optimal concentration. Illumina NovaSeq 6000 was used for Pair-End 151bp sequencing. Using software from Illumina (bcl2fastq), sequencing reads were assigned. An average of 95% of the bases achieved the accuracy of 99.9%.

***Polysome fractionation experiments***

Cells were seeded in 175 cm^2^ flasks and used for fractionation when they were 90% confluent. Before fractionation, cells were incubated in cycloheximide at 37 ^0^C for 10 minutes to freeze ribosomes on mRNA. After incubation, the cell pellets were collected, washed with PBS, split into two and re-suspended in hypotonic buffer supplemented with DMSF, EDTA-free protease inhibitor, cycloheximide, DTT, RNasin ribonuclease inhibitor and incubated on ice for 10 minutes. The cells were then lysed by adding 50 μl of 10% Triton X-100 and 50 µl of 10% Sodium deoxycholate and incubated on ice for 10 minutes. The concentration of RNA was measured and 1mg of total RNA subjected to the ultra-centrifugation. Due to the difference in weight, the actively translating mRNAs were separated from inactively translating mRNAs in 5% to 50% sucrose gradient by ultra-centrifugation at 35,000 x g for two hours at 4 ^0^C. The fractionation was done manually by adding 12 drops of lysate into each fraction. The peristaltic pump flow rate was set to be 400 μL per minute. The chart speed was 0.5 mm/sec and the sensitivity of UV monitor was 100 mV. The fractions were collected by fraction collector.

***Real time quantitative PCR***

The total volume of each reaction was 20 µl in 384-well plate. Each reaction contained 10 µL of 2x Power SYBR^®^ Green PCR Master Mix (Applied Biosystems), 0.5 μl of 10 µM forward and reverse primers, up to 6 µl of 10-fold diluted cDNAs and H_2_O. The sequences of primers used are shown in supplementary table 1. The relative quantity of each gene was determined using the comparative 2^-ΔΔCT^ method.

***Molecular cloning***

eIF4E amplicon was amplified by eIF4E-EcoR1-F and eIF4E-Xba1-R using *pCMV6-eIF4E* as the template. The sequences of the primers were shown in supplementary table 1. Restriction enzymes *EcoR*1 (NEB) and *Xba*1 (NEB) were used. The inert was cloned into *pcDNA3.1* to generate *pcDNA3.1-eIF4E WT*. QuikChange II Site-Directed Mutagenesis Kit (Agilent) was employed to generate eIF4E mutants to create *pcDNA3.1-eIF4E S209A* and *pcDNA3.1-eIF4E S209D*.

***siRNA***

eIF4E #siRNA1 (Dharmacon; J-003884-07; GAC GAU GGC UAA UUA CAU U); eIF4E #siRNA2 (Dharmacon; J-003884-08; CAU AUC CAG UUG UCU AGU A); ERα #siRNA1 (Dharmacon; J-003401-11; CCA GAC AGC GGG CAA AGU G); ERα #siRNA2 (Dharmacon; J-003401-12; GCG AGA AGC UCU GCA CGA G); FOXM1 #siRNA1 (Dharmacon; J-009762-05; CAA CAG GAG UCU AAU CAA G); FOXM1 #siRNA2 (Dharmacon; J-009762-06; GGA CCA CUU UCC CUA CUU U), MYC siRNA (Sigma; CGU CCA AGC AGA GGA GCA A) [1], Cyclin D1 siRNA (Sigma; AAC AAG CUC AAG UGG AAC CUG) [2] and the non-targeting control (siCtrl: D-001206-13).

***Cell viability test***

Clonogenic assay was performed by staining the cell colony was 0.01% of crystal violet.

***Western Blot***

The concentrations of proteins were determined by DC Protein Assay (BioRad) following manufacturer’s protocol. The following antibodies were used: ERα (HC-20, Santa Cruz, 1:1000), eIF2α (#9722, Cell Signalling Technology, 1:1000), eIF3H (#3413; Cell Signalling Technology, 1:2000), eIF4A (#2425; Cell Signalling Technology, 1:4000), eIF4B (#3592, Cell Signalling Technology, 1:1000), eIF4E (#9742, Cell Signalling Technology, 1:1000), p-eIF4E (S209) (#9741, Signalling Technology, 1:1000), eIF4G (#2498; Cell Signalling Technology, 1:1000), eIF6 (#3263, Cell Signalling Technology, 1:1000), 4E-BP1 (#9452, Cell Signalling Technology, 1:1000), anti-Flag (M2, Sigma, 1:3000), FOXM1 (C-20, Santa Cruz, 1:1000), Akt (#9272, Cell Signalling Technology, 1:1000) and β-tubulin (Santa Cruz, 1:1000).

***Immunohistochemistry***

Antigen was retrieved by heating in microwave in citrate buffer for 15 min and primary antibodies were diluted as 1:200 for eIF4E (Cell Signalling), 1:100 for ERα (Santa Cruz), and 1:50 for FOXM1 (Santa Cruz). Aperio ScanScope® system (Aperio technology) was used to assess staining of the carcinoma cells. The eIF4E score took into account the total score obtained from both cytoplasmic and nuclear staining. Median score for eIF4E was taken as cut off between high versus low expression. For ERα and FOXM1 expression only nuclear staining was considered, since ERα is a nuclear receptor and FOXM1 a transcription factor.

**References**

1 Reyes-Gonzalez JM, Armaiz-Pena GN, Mangala LS, Valiyeva F, Ivan C, Pradeep S *et al*. Targeting c-MYC in Platinum-Resistant Ovarian Cancer. *Molecular Cancer Therapeutics* 2015; 14: 2260-2269.

2 Yu ZR, Wang LP, Wang CG, Ju XM, Wang M, Chen K *et al*. Cyclin D1 induction of Dicer governs microRNA processing and expression in breast cancer. *Nature Communications* 2013; 4.
